# Profiling of MicroRNAs and Their Targets in Roots and Shoots Reveals a Potential MiRNA-Mediated Interaction Network in Response to Phosphate Deficiency in the Forestry Tree *Betula luminifera*

**DOI:** 10.3389/fgene.2021.552454

**Published:** 2021-01-28

**Authors:** Junhong Zhang, Yan Lin, Fangmin Wu, Yuting Zhang, Longjun Cheng, Menghui Huang, Zaikang Tong

**Affiliations:** State Key Laboratory of Subtropical Silviculture, School of Forestry and Bio-Technology, Zhejiang A&F University, Hangzhou, China

**Keywords:** abiotic stress, degradome, transcriptome, miRNA, Pi deficiency, *Betula luminifera*

## Abstract

Inorganic phosphate (Pi) is often lacking in natural and agro-climatic environments, which impedes the growth of economically important woody species. Plants have developed strategies to cope with low Pi (LP) availability. MicroRNAs (miRNAs) play important roles in responses to abiotic stresses, including nutrition stress, by regulating target gene expression. However, the miRNA-mediated regulation of these adaptive responses and their underlying coordinating signals are still poorly understood in forestry trees such as *Betula luminifera*. Transcriptomic libraries, small RNA (sRNA) libraries, and a mixed degradome cDNA library of *B. luminifera* roots and shoots treated under LP and normal conditions (CK) were constructed and sequenced using next-generation deep sequencing. A comprehensive *B*. *luminifera* transcriptome derived from its roots and shoots was constructed, and a total of 76,899 unigenes were generated. Analysis of the transcriptome identified 8,095 and 5,584 differentially expressed genes in roots and shoots, respectively, under LP conditions. sRNA sequencing analyses indicated that 66 and 60 miRNAs were differentially expressed in roots and shoots, respectively, under LP conditions. A total of 109 and 112 miRNA–target pairs were further validated in the roots and shoots, respectively, using degradome sequencing. Kyoto Encyclopedia of Genes and Genomes pathway enrichment analysis of differential miRNA targets indicated that the “ascorbate and aldarate metabolism” pathway responded to LP, suggesting miRNA-target pairs might participating in the removing of reactive oxidative species under LP stress. Moreover, a putative network of miRNA–target interactions involved in responses to LP stress in *B. luminifera* is proposed. Taken together, these findings provide useful information to decipher miRNA functions and establish a framework for exploring P signaling networks regulated by miRNAs in *B. luminifera* and other woody plants. It may provide new insights into the genetic engineering of high use efficiency of Pi in forestry trees.

## Introduction

Phosphate (P), one of the essential macronutrients in plants, is often not readily accessible to plants because of the low availability of inorganic phosphate (Pi), the major acquired form of P, in the soil (Marschner, 1995). Therefore, plants are frequently exposed to conditions of Pi limitation. To maintain plant productivity, large quantities of Pi fertilizers are applied, which is not only expensive but also harmful to the environment (Cordell et al., 2009). Genetic engineering of plants with enhanced Pi starvation tolerance is a promising approach to address these challenges. Therefore, a great deal of effort has been directed toward understanding the mechanisms of plant adaptation to Pi deficiency.

Plants have evolved the ability to acclimatize to Pi deficiency by triggering the Pi starvation response (PSR), which includes a series of morphological, physiological, and biochemical/metabolic adaptations. For example, extending the root’s surface area for phosphate absorption underlies the morphological adaptations of Pi starved plants (Vance et al., 2003); increased synthesis of anthocyanins (Takahashi et al., 1991) and the synthesis of acid phosphatase enzymes (APases) is a universal response by plants to Pi deficiency (Duff et al., 1994; Tran et al., 2010). The PSR is partially achieved by the coordinated induction of hundreds of Pi starvation-inducible (PSI) genes, which increase Pi use efficiency and enhance Pi acquisition from the environment (Lin et al., 2009; Nilsson et al., 2010). These PSI genes are involved in Pi uptake, allocation, or remobilization, and include transcription factors, signaling molecules, and other upstream regulators (Lin et al., 2009; Zeng et al., 2016). Pi uptake by roots occurs mainly through Pi transporters, encoded by Phosphate Transporter1 (*PHT1*), of which PHT1;1 and PHT1;4 play major roles in acquiring Pi from the soil in *Arabidopsis* (Shin et al., 2004). Phosphate1 (*PHO1*) is located in root stelar cells, and participates in Pi transfer from roots to shoots (Hamburger et al., 2002). Under Pi starvation, WRKY6 and WRKY42 are degraded *via* 26S proteasome-mediated proteolysis (Chen et al., 2009; Su et al., 2015). Phosphate Response Ubiquitin E3 Ligase1 (*PRU1*) modulates Pi homeostasis by regulating the abundance of WRKY6 in response to low-Pi (LP) stress in *Arabidopsis* (Ye et al., 2018). The recent development of next-generation sequencing technology has facilitated the studies of the transcriptome at a whole-genome scale with single-base resolution (Trapnell et al., 2012), triggering a large number of Pi-responsive genes have been identified in several plants. For example, Secco et al. (2013) analyzed 126 paired-end RNA sequencing (RNA-Seq) libraries, and presented comprehensive spatiotemporal transcriptome analysis of responses to Pi stress in rice, revealing a large number of potential key regulators of Pi homeostasis in plants. In soybean, a total of 1,612 genes were differentially expressed in roots after exposure to Pi deficiency for 7 days (Zeng et al., 2016), and 2,055 genes exhibited differential expression patterns between Pi sufficient and deficient conditions in nodule (Xue et al., 2018). White lupin (*Lupinus albus*) has evolved unique adaptations for growth in Pi-deficient soil, and 2,128 sequences were shown to be differentially expressed in response to Pi deficiency (O’Rourke et al., 2013). Huang et al. (2019) identify 237 genes differentially expressed in the vasculature of *Plantago major* upon 24 h of Pi deficiency using RNA-Seq, of which only 27 have been previously identified to be specifically expressed in the vasculature tissues in other plant species.

Recent studies have also emphasized the importance of posttranscriptional mechanisms in the control of PSI gene expression. There is accumulating evidence that microRNAs (miRNAs) play critical roles in sensing Pi abundance, controlling Pi uptake and phloem-mediated long-distance transport, and Pi homeostasis (Hsieh et al., 2009; Zeng et al., 2013). MiRNAs negatively regulate gene expression by modulating both mRNA degradation and translational inhibition (Jones-Rhoades et al., 2006). Their expression profiles are significantly altered under conditions of stress, suggesting that attenuated plant growth and development under stress may be controlled by stress-responsive miRNAs (Sunkar et al., 2012). Recent studies have shown that abiotic stresses induce aberrant expression of many miRNAs, suggesting that miRNAs may be new targets for genetically improving plant tolerance to certain types of stress (Zhang, 2015). Mounting evidence shows that miRNAs play key roles in regulating nutrient metabolism in plants (Liang et al., 2015). Many miRNAs are involved in plant responses to nutrient stresses by acting as systemic signals to coordinate physiological activities that help plants to respond and survive these stresses (Zeng et al., 2013). A series of Pi starvation-responsive miRNAs were identified *via* high-throughput sequencing of small RNAs (sRNAs), which mediate the regulation of Pi uptake, transport, and assimilation in plants by targeting a set of genes (Kumar et al., 2017). For example, Hsieh et al. (2009) reported that the expression of miR156, miR399, miR778, miR827, and miR2111 was induced, whereas the expression of miR169, miR395, and miR398 was repressed upon Pi deprivation in *Arabidopsis* using sRNA sequencing. Xu et al. (2013) found 25 miRNAs were induced and 11 miRNAs were repressed by Pi starvation in soybean using sRNA sequencing, and 154 target genes of miRNAs were predicted *via* degradome sequencing. The induction of miR399 and miR398 and the downregulation of miR156, miR159, miR160, miR171, and miR2111 were observed under Pi deficiency in alfalfa (Li et al., 2018). Several conserved families, such as miR156, miR159, miR166, miR169, miR319, miR395, miR397, miR398, miR399, and miR827 have been reported to be commonly responsive to Pi starvation in various plant species, suggesting that these miRNAs are coordinately involved in the conserved Pi signaling pathways in plants (Sunkar et al., 2012). Of these miRNAs, miR399 has attracted the most attention. miR399 is induced by Pi starvation and regulates Pi homeostasis through Pi acquisition, allocation, and remobilization by downregulating its target gene *PHO2* (Fujii et al., 2005; Chiou et al., 2006; Pant et al., 2008). Phosphate Starvation Response 1 (*PHR1*), PHR1-LIKE 1 (*PHL1*), *MYB2*, and several other Pi starvation-responsive genes act upstream to induce increased miR399 expression (Zeng et al., 2013). Hsieh et al. (2009) suggest that miR169 members are downregulated by LP, and some have PHR1 binding sites in their upstream regions. Li et al. (2016) also show that the promoter regions of miR169c/o/q harbored PHO-like binding sites and/or W-box elements in soybean. miR827 is also specifically induced by Pi starvation and plays a critical role in regulating Pi homeostasis by downregulating its target gene Nitrogen Limitation Adaptation (*NLA*) in *Arabidopsis* (Hsieh et al., 2009; Kant et al., 2011). miR827 is regulated by *OsPHR2* (an ortholog of *AtPHR1*), and overexpression of miR827 leads to increased Pi contents in leaves by decreasing Pi remobilization from old to young leaves (Wege et al., 2016). Wheat TaemiR408 was induced upon Pi starvation, and tobacco lines with TaemiR408 overexpression showed increased P accumulation upon Pi deprivation, suggesting the role of miR408 in improving plant Pi uptake (Bai et al., 2018). Khandal et al. (2020) show a regulatory role for the miR397b-*LAC2* module in root lignification during phosphate deficiency. However, some Pi-responsive miRNAs are species-specific and may participate in exclusive pathways by regulating specific target genes. It suggests that identification of species-specific miRNA and the corresponding targets would be meaningful for improving the ability to adapt to Pi deficiency.

*Betula luminifera* is a broadleaf tree species belonging to the Betulaceae family that is indigenous to China and naturally distributed in more than 14 provinces as part of the subtropical forest ecosystems in China (Huang et al., 2014; Zhang J.H. et al., 2016). Its desirable wood properties and fast-growing features make it the most common afforestation species in southern China. However, *B. luminifera* frequently encounters conditions of P limitation. Although the total P content in soil is generally high, the abundance of the orthophosphate form (Pi) available to plants is often limited in acidic soil due to adsorption by soil particles, precipitation, or conversion to organic forms (Zeng et al., 2016). In the present study, we aimed to uncover the complex transcriptional and posttranscriptional responses of *B*. *luminifera* to Pi stress, which might aid the development and selection of trees with increased P use efficiency. First, we generated a comprehensive and integrated dataset that provides insight into the molecular responses of the *B*. *luminifera* transcriptome using next-generation RNA-Seq. Second, to investigate the miRNA-regulated network response to Pi deficiency, deep sequencing of the small RNAome and degradome were performed to determine the responsive miRNAs and the gene targets of specific miRNAs. Then, these analyses were integrated to elucidate the molecular network of the posttranscriptional response to Pi deficiency in *B*. *luminifera*. These results will enable future researchers to further elucidate transcriptional and posttranscriptional responses of *B*. *luminifera* to Pi deficiency, which may help to create plants proficient to offset Pi deficiency.

## Materials and Methods

### Plant Materials and Growth Conditions

The *B. luminifera* clone designated G49^#^ was propagated *in vitro* in the laboratory at 25 ± 2°C under fluorescent light with a 16-h photoperiod, and the seedlings were grown in Murashige and Skoog (MS) medium without growth regulators. Two-month-old tissue culture seedlings of G49^#^ were subsequently planted in pots with washed and sterilized sand, with three holes at the bottom of the pot for drainage. Then, seedlings were grown in an incubator with a light intensity of 150 μmol/m^2^/s and temperature of 25/20°C (day/night), which were irrigated with modified liquid MS medium containing normal Pi for 2 weeks, and irrigated with deionized water for 2 weeks. Then, 54 uniformly developed plants were divided into two groups, and a completely randomized block design with three replicates was used. For the control (CK) treatment, MS medium contained 1.25 mM KH_2_PO_4_, without KH_2_PO_4_ (the only source of Pi) for the phosphate deficiency treatment (i.e., LP), and adjusted the K^+^ concentration accordingly by adding K_2_SO_4_ (Jain et al., 2009; Zeng et al., 2016). The pH of the medium was adjusted to 5.7, and medium was used for irrigation every day. Shoots and roots were harvested after days 3, 7, and 15 of the phosphate deficiency treatment.

### Determination of P Concentration, Anthocyanin, and Acid Phosphatase

Fine powder (∼100 mg dry weight) was digested in a mixture of 5 mL 98% H_2_SO_4_ and 1 mL H_2_O_2_, and the P content was determined spectrophotometrically at 700 nm based on the molybdenum blue method (Sun et al., 2012). The anthocyanin concentrations in the shoots under Pi starvation were analyzed spectrophotometrically according to the method of Lei et al. (2011). APase activity in the roots and shoots were analyzed using the modified *p*-nitrophenyl phosphate (NPP) method (Liu et al., 2004).

### RNA Extraction, Library Construction, and Sequencing

Shoots and roots were sampled after 7 days of the phosphate deficiency treatment, which were used to RNA sequencing, according to Zeng et al. (2016). Specifically, total RNA was isolated using Trizol reagent (Invitrogen, Carlsbad, CA, United States). Total RNA quality and quantity were analyzed using a Bioanalyzer 2100 and RNA 6000 Nano LabChip Kit (Agilent Technologies, Santa Clara, CA, United States) with RNA integrity number (RIN) > 7.0. Under CK and LP treatment, each mixed from three biological replicates were used for library construction and sequencing, thus root RNA was used to construct a root library, and equal quantities of RNA from stems and leaves were pooled to generate a shoot library. For transcriptome sequencing, approximately 10 μg of total RNA was subjected to poly(A) mRNA isolation using poly-T oligo-attached magnetic beads (Invitrogen). Following purification, the mRNA was fragmented into small pieces using divalent cations under elevated temperature conditions. The cleaved RNA fragments were then reverse transcribed to generate the final cDNA library using the mRNA-Seq sample preparation kit (Illumina), and the average insert size for the paired-end libraries was 300 (±50) bp. Paired-end sequencing was then performed on an Illumina Hiseq2500 at the facilities of LC-BIO (Hangzhou, China).

Approximately 1 μg of total RNA was used to construct sRNA library using a TruSeq Small RNA Sample Prep Kit in accordance with the manufacturer’s protocol (Illumina, San Diego, CA, United States). Single-end sequencing (36 bp) was then performed on an Illumina HiSeq2500 at the facilities of LC-BIO (Hangzhou, China).

### Sequence Data Analysis

For transcriptome data, prior to assembly, the low-quality reads, including reads containing sequencing adaptors and sequencing primers, nucleotides with quality score <20 were removed, and cleaned sequences were obtained. Sequencing reads were assembled *de novo* using Trinity software with the default parameters. All unigene sequences were compared against the protein databases (Nr, SwissProt, KEGG, COG) using BLASTx (*E*-value < 0.00001). Gene ontology (GO) functional annotation was obtained from SwissProt annotation. To investigate the expression level of each unigene in all samples, all paired-end reads for each sample were aligned back to the final assembly using Perl scripts in Trinity with the default parameters. The alignment produced digital expression levels for each contig and these were then normalized by RESM-based algorithm using Perl scripts in the Trinity package to obtain RPKM values. Based on expression levels, significantly differentially expressed mRNAs (DE mRNAs) among the four samples were identified by *P* ≤ 0.05 and log_2_ fold-change (log_2_FC) ≥ 1. Clustering of DE mRNAs was performed using the common Perl and R scripts. DE mRNAs were identified using the GOseq R package (Young et al., 2010), which is based on the Wallenius non-central hyper-geometric distribution, with the threshold *P* ≤ 0.05. In addition, Kyoto Encyclopedia of Genes and Genomes (KEGG) pathway enrichment analysis of the DE mRNAs was performed using KOBAS (2.0) with false discovery rate (FDR) ≤ 0.05 (Mao et al., 2005).

For sRNA data, the raw reads were subjected to the Illumina pipeline filter (Solexa 3.0), and the dataset was further processed using an in-house program (ACGT101-miR; LC Sciences, Houston, TX, United States) (Li et al., 2010) to remove adaptors, junk, regions of low complexity, common RNA families (rRNA, tRNA, snRNA, and snoRNA), and repeats. As described in our previous report (Zhang J.H. et al., 2016), the filtered sequencing reads were compared to known miRNAs in miRBase 22.0^1^ (Kozomara and Griffiths-Jones, 2013), and the remaining sRNA sequences were used to identify potential novel miRNAs based on the criteria for the miRNA definition described by Meyers et al. (2008). miRNA differential expression based on normalized deep-sequencing counts was analyzed by selectively using Fisher’s exact test, with the significance threshold set to 0.05. The resulting network was visualized by Cytoscape 3.7.0 (Shannon et al., 2003).

### Degradome Library Construction and Target Identification

The putative target genes in *B. luminifera* were predicted using TargetFinder software. Mismatched pairs or single nucleotide bulges were each scored as one and G:U pairs were scored as 0.5. Mismatched and G:U pair scores were doubled within the core segment (nucleotide pairs at positions 2–13) and score ≤ 4 (Jones-Rhoades and Bartel, 2004). The clean sequences of the high-throughput degradome sequencing were compared to the integrated transcriptome dataset. Then, identification and classification of the sliced miRNA target categories were performed according to the CleaveLand 3.0 pipeline (Charles Addo-Quaye et al., 2008).

To better elucidate the potential miRNA regulatory roles, we subjected all identified target genes to GO analysis. GO annotations of all targets were performed using AgriGO^2^ (Du et al., 2010). Differentially expressed miRNAs (DE miRNAs) with their target lists were investigated for cognate mRNA targets in their respective DE mRNA list to delineate miRNA–mRNA functional interactions using ACGT101-CORR1.1, as described previously (Zhang G. et al., 2016).

### Validation of MiRNA Target Genes

5′RLM-RACE verification was performed to further validate the target genes, using the GeneRacer Kit (Invitrogen) as described previously (Sunkar et al., 2005). Total RNA was extracted as described above, and the primers used are listed in Supplementary Table 1. The PCR products were cloned, and at least 10 independent clones were sequenced to analyze the cleavage sites.

Transient overexpression of *MIR169* precursors in *B. luminifera* leaves was conducted to validate the corresponding target genes. Briefly, the agrobacterium GV3101 containing *35S:MIR169a* and *35S:MIR169c* was overnight propagated, collected, and resuspended in mixture containing 10 mM MgCl_2_, 10 mM MES, pH 5.6, and 150 μM acetosyringone, respectively. Then agrobacterium suspension was injected into three-week-old *B. luminifera* leaves. Leaves were harvested after 3 days of injection, then the expression levels of *NFYA1* and *NFYA10* were determined by qRT-PCR.

### Verification by qRT-PCR

Total RNA was extracted from roots, stems, and leaves after 3, 7, and 15 days under CK and LP conditions as described above. RNA from roots was used to detect the expression patterns of genes and miRNAs, and equal quantities of RNA from stems and leaves were pooled to monitor their expression levels. qRT-PCR of genes was performed using a PrimeScript RT Reagent Kit and SYBR Premix EX Taq Kit (TaKaRa Biotechnology), in accordance with the manufacturer’s instructions. The expression levels of target genes were normalized relative to that of Malate Dehydrogenase (*MDH*, KP245810) and α-tubulin (*TUA*, KP245813) in roots and shoots, respectively. The primers of target genes and their reference genes are listed in Supplementary Table 2. Reactions were performed using a CFX96 Real-time PCR Detection System (Bio-Rad, Hercules, CA, United States) with three replicates per sample. After PCR, dissociation curves and 2% agarose gel electrophoresis were performed to verify the specificity of amplification.

miRNA qRT-PCR was performed using the RNA as the template and Mir-X miRNA qRT-PCR SYBR Kits (Takara-Clontech, Shiga, Japan), in accordance with the manufacturer’s instructions. The forward primers of selected miRNAs were designed based on the mature miRNA sequence, as listed in Supplementary Table 3, and the reverse primer was provided with the kit. All expression levels were normalized relative to that of U6 (forward primer: 5′-TCGGGGACATCCGATAAAATTGGAA-3′, reverse primer: 5′-GGACCATTTCTCGATTTATGCGTGTCA-3′), and the reactions were performed as described above.

### Statistical Analysis

Statistical tests were performed using SPSS 17.0 (SPSS Inc., Chicago, IL, United States). Data were considered to be significantly different at *P* < 0.05 in the *t*-test. Correlation analysis between the miRNAs and target genes was conducted using bivariate correlation in SPSS.

## Results

### Pi Deprivation Alters Physiological Characteristics

**FIGURE 1:**
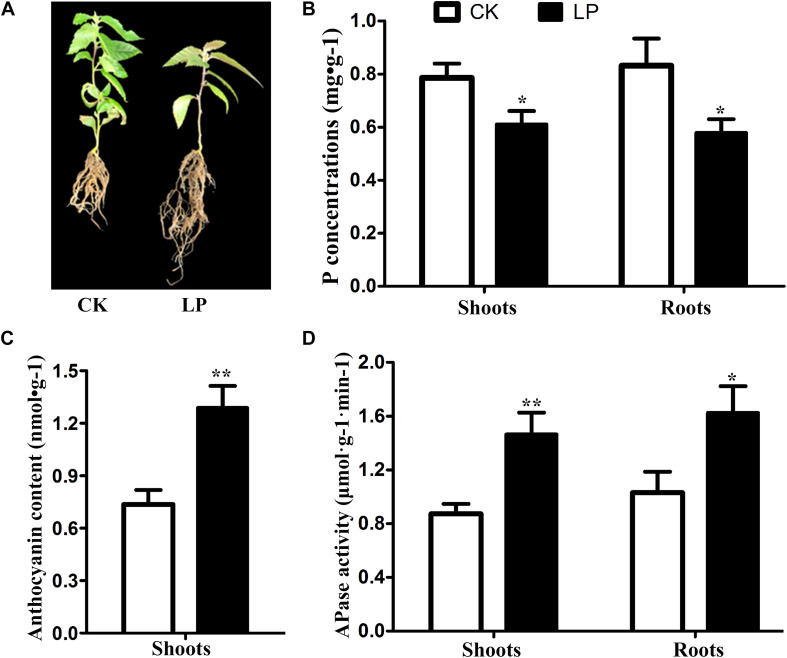
Changes in phenotype **(A)**, P concentration **(B)**, anthocyanin content **(C)**, and APase activity **(D)** in *B*. *luminifera* after 7 days of Pi starvation. Bars indicate means ± SE (*n* = 3). *P*-values were obtained from *t*-tests between LP and CK conditions. **P < 0.01; *P < 0.05.

After day 7 of Pi starvation, the P concentration was decreased significantly both in shoots and roots, and the anthocyanin content in shoots was increased significantly. However, APase activity in roots and shoots increased significantly under Pi deprivation. The seedlings of *B. luminifera* showed attenuated growth and increased root biomass after day 15 of Pi starvation (Figure 1).

### The Pi Deficiency-Responsive Transcriptome in *Betula luminifera*

**FIGURE 2:**
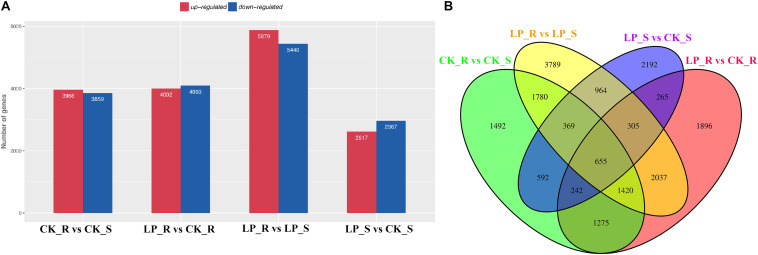
Expression profiling of –Pi-responsive DE mRNAs in *B*. *luminifera*. **(A)** The numbers of DE mRNAs in roots and shoots after 7 days of Pi starvation compared with control plants were determined by *P* ≤ 0.05 and log_2_FC ≥ 1. Red and blue columns represent upregulated and downregulated DE mRNAs, respectively. **(B)** Venn diagrams showing the numbers of overlapping and specific DE mRNAs obtained across four comparisons. R, roots; S, shoots.

Overall, more than 216 million raw reads were obtained (GSM4105637–GSM4105640). After removing reads that failed to meet quality control standards, valid reads were used for *de novo* assembly, resulting in 154,340 transcripts and 76,899 unigenes. Unigene expression levels were calculated using transcripts per million (TPM). Under conditions of Pi starvation, 4,002 DE mRNAs were upregulated and 4,093 DE mRNAs were downregulated in roots, whereas 2,617 and 2,967 DE mRNAs were upregulated and downregulated, respectively, in shoots (Figure 2A). A total of 1,467 DE mRNAs were common to roots and shoots (Figure 2B).

Gene ontology enrichment analysis showed that 26 and 38 GO terms were significantly enriched in roots and shoots, respectively, at a threshold of *P* < 0.001. In roots, the three most enriched GO terms were “nucleus” (GO: 0005634), “chloroplast” (GO:0009507), and “response to salt stress” (GO:0009651). However, the three most enriched GO terms in shoots were distinct from those enriched in roots, i.e., “extracellular region” (GO:0005576), “DNA binding” (GO:0003677), and “cell cycle” (GO: 0007049).

Kyoto Encyclopedia of Genes and Genomes enrichment analysis showed that 10 and 23 terms were significantly enriched in roots and shoots, respectively, with the threshold of *P* < 0.05 (Supplementary Figure 1). In roots, the most enriched KEGG terms were “plant hormone signal transduction” (ko04075), “glycosphingolipid biosynthesis – lacto and neolacto series” (ko00601), and “ubiquitin mediated proteolysis” (ko04120) in roots. A total of 229 DE mRNAs involved in signaling of various hormones, including auxin, ethylene, abscisic acid (ABA), and jasmonic acid, were identified in Pi-deficient roots, of which 126 DE mRNAs were downregulated. Nineteen auxin-related genes were found to be responsive to Pi starvation, with six genes showing repression and 13 genes showing induction. However, the most enriched KEGG terms were “phenylpropanoid biosynthesis” (ko00940), “flavonoid biosynthesis” (ko00941), and “starch and sucrose metabolism” (ko00500) in shoots (Supplementary Figure 1B). Totals of 121 and 46 DE mRNAs were involved in phenylpropanoid and flavonoid biosynthesis, respectively, and more than half of the DE mRNAs were repressed under conditions of Pi starvation.

To validate the expression levels identified by RNA-seq, 16 differentially expressed genes were arbitrarily selected for expression analyses after 3, 7, and 15 days of LP treatment using qRT-PCR. The qRT-PCR results showed that the expression levels of the examined genes, with the exception of *PER57* in shoots, and *WRKY41* in roots, exhibited similar trends as those seen the RNA-seq results after 7 days of LP treatment (Supplementary Figure 2).

### Analysis of sRNA Populations Under Conditions of Pi Deficiency

A total of approximately 47 million raw reads were obtained from sRNA libraries (GSM4105633–GSM4105636). After removing reads that failed to meet quality control standards, there were 31,980,253 redundant reads (10,503,545 non-redundant reads). The sequences were aligned to known RNAs in RFam, mRNA, Repeat-repbase, and other known RNA sequences; 16,203,000 redundant reads (9,688,171 non-redundant sequences) were processed for further analysis.

**FIGURE 3:**
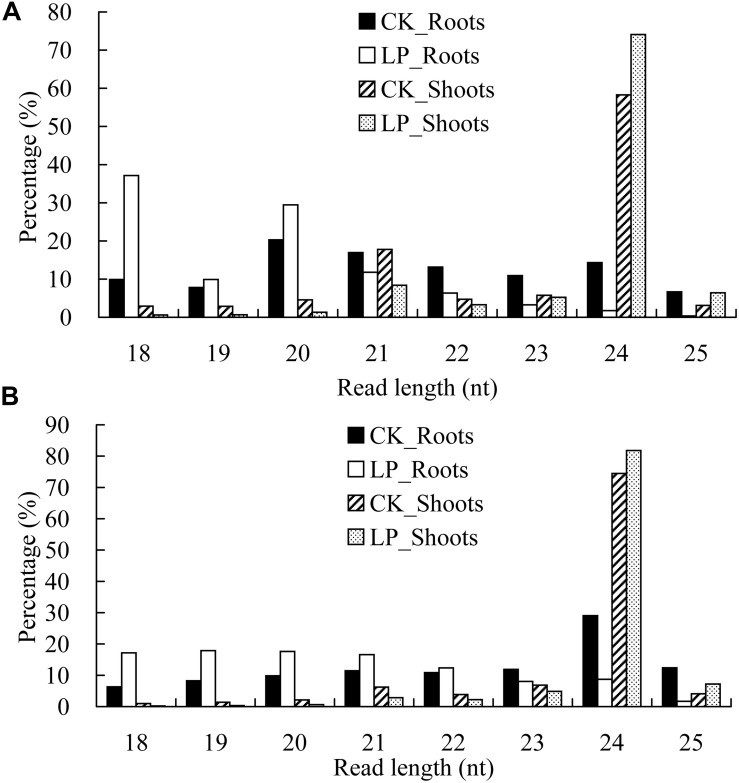
Global profiling of processed small RNAs (sRNAs) in *B*. *luminifera*. **(A)** Frequencies of processed sRNAs are expressed as percentages of the total number of redundant sequences. **(B)** Frequencies of processed sRNAs are expressed as percentages of the total number of non-redundant sequences. Four samples including CK_Roots (black bars), LP_Roots (white bars), CK_Shoots (striped bars), and LP_Shoots (dotted bars) in roots and shoots under normal and phosphate-deficient (–Pi) conditions.

For the redundant sequences, there were major peaks at 24 nucleotides (nt) in the shoot libraries. Unexpectedly, a major peak at 21 nt was observed in the roots under LP conditions (Figure 3A). For the non-redundant sequences, an overwhelming peak was seen at 24 nt in the shoot sRNA libraries (Figure 3B), while the sRNA length was preferentially 18–21 nt under LP conditions. Further analysis showed that tRNA ratios with sizes of 18 and 19 nt were significantly increased in LP-treated roots, especially for 18 nt, whereas the corresponding ratios were significantly decreased in LP-treated shoots (Supplementary Table 4).

### Identification of Pi Deficiency-Responsive MiRNAs in *Betula luminifera*

A total of 164 known miRNAs from 56 families were identified based on a homology search for known miRNAs using miRBase (release 22.0) as a reference set. Of these miRNAs, 82 were identified unambiguously, as these miRNAs had corresponding precursor sequences, whereas 82 miRNAs without corresponding precursor sequences were identified ambiguously (Supplementary Table 5). These miRNAs were named using the initials of the species (*blu* for *B. luminifera*) followed by the number (and letter when applicable) assigned to the same miRNA in other species. Furthermore, 19 novel *MIRNA* genes were identified with both 5′ and 3′ arms, and 21 were considered as candidate miRNAs with 5′ or 3′ arms (Supplementary Table 6).

Totals of 66 miRNAs were differentially expressed in Pi-deficient roots, including 20 that were upregulated and 20 that were downregulated, as well as 9 and 17 miRNAs that were specific to LP-treated and control roots, respectively (Supplementary Table 7 and Supplementary Figure 3A). Sixty miRNAs were differentially expressed in Pi-deficient shoots, consisting of 20 that were upregulated and 33 that were downregulated, and five and two miRNAs were specific to LP-treated and control shoots, respectively (Supplementary Table 8 and Supplementary Figure 3B). Of these DE miRNAs, 17 shared the same expression trends between roots and shoots, whereas six miRNAs showed the opposite expression patterns between roots and shoots. Furthermore, 80 of the DE miRNAs were Pi-responsive and tissue-specific (43 and 37 in roots and shoots, respectively). Specifically, in the −Pi roots, miR1511a/b, miR399c, miR397a/b/c, miR398a/b, miR858, and miR169b were induced, whereas miR319b-5′, miR530a, miR169c, miR396b, and miR164 were repressed. In the −Pi shoots, miR397a/b, miR398b, miR156i, miR408a, miR396d-3′, and six novel miRNAs were upregulated, whereas miR319a-5′, miR169a/b, miR159c, miR395b, miR828b, and miR172a were downregulated.

**FIGURE 4:**
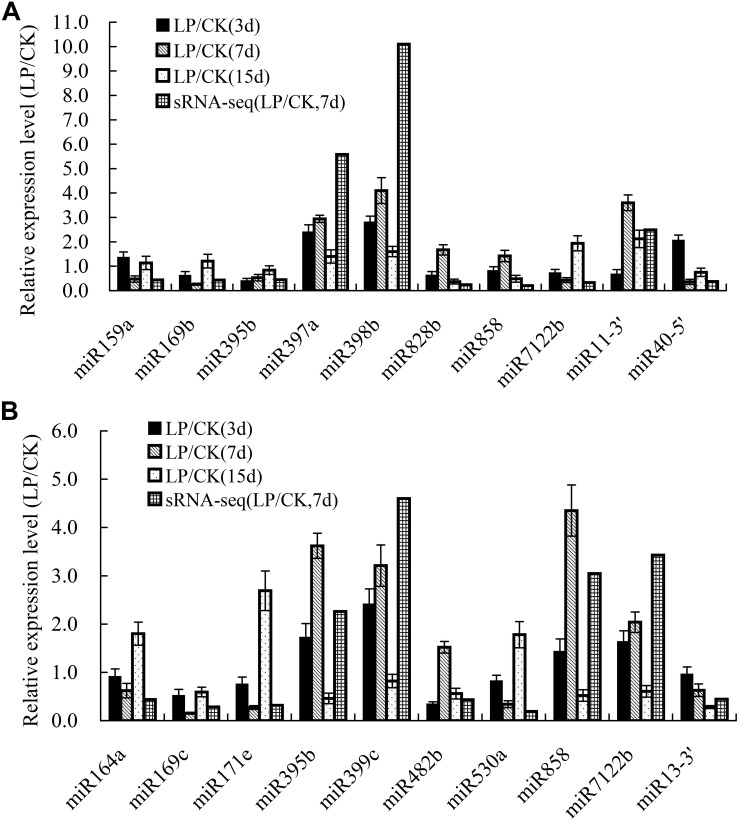
Validation of the expression profiles of miRNAs in *B. luminifera* shoots and roots identified by sRNA-seq using qRT-PCR. **(A)** Relative expression of ten LP-responsive miRNA in shoots after 3, 7, and 15 days of LP treatment; **(B)** Relative expression of ten LP-responsive miRNA in roots after 3, 7, and 15 days of LP treatment. Expression level is represented by the ratio of LP treatment to CK. The value of relative expression level above one indicates that miRNAs are upregulated by Pi starvation, and the value below one shows that miRNAs are downregulated by Pi starvation. The experiments were repeated three times. Error bars indicate standard deviation.

To validate the expression patterns identified by sRNA sequencing, 20 differentially expressed miRNAs after 3, 7, and 15 days of Pi deficiency were arbitrarily selected for expression analyses using qRT-PCR. The qRT-PCR results showed that the expression of most examined miRNAs, except for miR828b and miR858 in shoots, and miR482b in roots, exhibited similar trends after 7 days of Pi deficiency as those revealed by sRNA-seq (Figure 4).

### Target Genes Regulated by DE MiRNAs

In roots, 579 unigenes were predicted as targets of 60 DE miRNAs using the TargetFinder software, consisting of 610 miRNA–target pairs (Supplementary Table 9), whereas 407 unigenes were predicted as targets of 51 DE miRNAs in shoots, consisting of 461 miRNA–target pairs (Supplementary Table 10). Some miRNAs were predicted to target multiple genes, exemplified by one novel miRNA (blu-miR40-5p) that had 112 potential target genes. To validate these target genes, a mixed degradome library was sequenced, yielding 45,697,220 raw fragments, of which 45,437,576 reads were mapped to referenced transcriptomic data; thus 54,744 transcripts were processed to target identification. Therefore, 109 and 112 miRNA–target pairs were further validated in the roots (Supplementary Table 9) and shoots (Supplementary Table 10), respectively. The identified targets were further classified into five categories (0–4) according to the relative abundance of tags at the target sites. For representative miRNAs of the five categories and their corresponding targets, red lines indicate the cleavage site of each transcript in Supplementary Figure 4. These targets of known miRNAs were mainly transcription factors (Table 1), suggesting that these miRNAs may play important roles in gene regulation. In addition, many target genes involved in specific pathways were also identified, for example, ATP SULFURYLASE 1 (*APS1*) by miR395b, and six *LAC* genes by miR397 (Table 1). We also identified a series of novel target genes that were targeted by isomiRNAs with lengths of 18 or 19 nt, which were only differentially expressed in roots, including a PHOSPHOLIPASE D DELTA (*PLDD*) and one unknown gene by miR399c (19 nt), seven targets including *WRKY24* by miR395c-5p (18 nt), as well as seven targets by miR482-5′ (Supplementary Table 9).

**TABLE 1:** Main target genes of DE miRNAs in *B*. *luminifera*.

miRNA	Targets	miRNA	Targets
miR156	*SPL14*	miR319	Two *TCP* genes
miR159	*GAMYB*	miR396	Four *GRF* genes
miR160	*ARF18*	miR536, blu-miR40-5p	Two zinc finger protein genes
miR164	*NAC21*	miR395b	*APS1*
miR166	five *HD-ZIP* genes	miR397	Six laccases
miR169	two *NFYA* genes	miR482b	Two disease resistance proteins
miR171	*SCL6*	miR828, miR858	Six *MYB* genes
miR172	three *AP2* genes		

**FIGURE 5:**
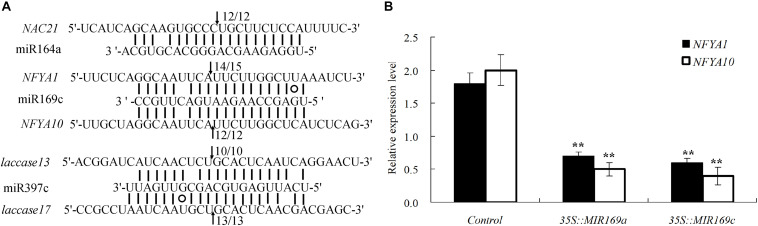
Validation of miRNA target genes. **(A)** Validation of five miRNA targets by 5′ RACE. Matches between miRNAs and their corresponding target genes are indicated by straight lines. G:U wobbles are represented by circles. Arrows indicate cleavage sites, and the clone frequencies are shown. **(B)** Two *NFYA* genes were validated by transiently overexpressing *BlMIR169a* and *BlMIR169c* in *B. luminifera* leaves.

We then performed 5′RLM-RACE for five of the identified miRNA targets for validation; one target of miR164, two potential targets of miR169, and two targets of miR397 were confirmed (Figure 5A). Furthermore, two *NFYA* genes were cleaved by miR169, as shown in a transient assay, in which the relative expression levels of *NFYA1* and *NFYA10* was decreased in the *B. luminifera* leaves transiently overexpressing *BlMIR169a* and *BlMIR169c* (Figure 5B).

Gene ontology enrichment analysis of targets for DE miRNAs revealed that 11 categories including “oxidoreductase activity, oxidizing metal ions,” “transcription factor activity, sequence-specific DNA binding” and “ATP binding” pathways responded to −Pi stress in roots; 17 GO categories, such as “transcription factor activity, sequence-specific DNA binding,” and “DNA binding,” and “cell differentiation” were enriched for targets of DE miRNAs in shoots (Supplementary Table 11). KEGG pathway enrichment analysis showed that “ascorbate and aldarate metabolism” was enriched for targets of DE miRNAs in both of roots and shoots, whereas “circadian rhythm – plant” was specifically enriched in shoots (Supplementary Table 12).

### Correlation Between DE MiRNAs and DE mRNAs of *Betula luminifera* in Response to LP Treatment

To determine the complex relationship between miRNA regulation and transcriptional response to Pi starvation, miRNA–mRNA interactions were predicted using sRNA-Seq, RNA-Seq, and degradome-Seq data. A total of 109 miRNA–target pairs were analyzed in roots, involving 38 DE miRNAs and 101 target genes validated by degradome-Seq. Thus, 30 pairs, including 14 DE miRNAs and 28 DE mRNAs, were identified, of which 14 miRNA–mRNA pairs showed opposite expression patterns under LP conditions (Table 2). For example, miR169c was downregulated significantly with log_2_FC of −1.81, and the target gene *NFYA10* was significantly induced with log_2_FC of 2.30 under –Pi conditions, which was predicted to participate in aminoacyl-tRNA biosynthesis (ko00970).

**TABLE 2:** Correlation analysis between differentially expressed miRNAs and their target genes under low phosphate conditions.

miR_name	Log2(FC)	Gene_ID	Annotation	log2(FC)
**In roots**				
blu-miR40-5′	-inf ^*a*^	TRINITY_DN23871_c0_g5	L484_015979 (*Morus notabilis*)	2.75
		TRINITY_DN25037_c1_g1	E3 ubiquitin-protein ligase MIEL1-like (*Malus domestica*)	2.71
		TRINITY_DN29288_c2_g1	Unknown	2.55
miR169b	1.07	TRINITY_DN26121_c0_g1	LOC109003207 (*J. regia*)	–2.61
miR169c	−1.81	TRINITY_DN23675_c2_g2	*NFYA1* (*J. regia*)	3.14
		TRINITY_DN24688_c1_g2	*NFYA10* (*J. regia*)	2.30
miR171e	−1.64	TRINITY_DN28131_c2_g2	*SCL27* (*J. regia*)	1.42
miR172e-5′	−1.16	TRINITY_DN27324_c1_g1	LOC108991439 (*J. regia*)	1.65
miR319b-5′	−3.04	TRINITY_DN28715_c2_g3	Putative mitochondrial protein (*Glycine soja*)	1.11
miR395c-5′	-inf^a^	TRINITY_DN24269_c0_g5	Transmembrane 9 superfamily member 8-like (*J. regia*)	1.04
miR403b	1.74	TRINITY_DN23405_c0_g6	LOC108990185 (*J. regia*)	–1.18
miR482b	−1.21	TRINITY_DN22938_c0_g10	Unknown	3.18
miR482b-5′	inf^*b*^	TRINITY_DN26208_c1_g4	Serine/threonine-protein kinase At5g01020-like (*J. regia*)	–1.76
miR7122b	1.78	TRINITY_DN27260_c2_g9	Pentatricopeptide repeat-containing protein At1g62914 (*Nelumbo nucifera*)	–1.01
**In shoots**				
blu-miR40-5′	−1.41	TRINITY_DN25037_c1_g1	E3 ubiquitin-protein ligase MIEL1-like (*M. domestica*)	1.92
blu-miR40-5′	−1.41	TRINITY_DN29286_c0_g3	Sucrose synthase 1 (*B. luminifera*)	1.50
miR159a	−1.18	TRINITY_DN24093_c0_g4	Transcription factor GAMYB (*J. regia*)	1.25
miR159b	-inf^a^	TRINITY_DN24093_c0_g4	Transcription factor GAMYB (*J. regia*)	1.25
miR319b-5′	−2.28	TRINITY_DN28715_c2_g3	Putative mitochondrial protein (*G. soja*)	1.77
miR397a	2.48	TRINITY_DN26138_c0_g6	Laccase 1 (*B. platyphylla*)	–2.67
miR397a	2.48	TRINITY_DN27015_c0_g1	Laccase-17-like (*J. regia*)	–1.54
miR397a	2.48	TRINITY_DN26934_c1_g1	Laccase-17-like (*J. regia*)	–1.83
miR397a	2.48	TRINITY_DN28629_c3_g4	Laccase-7-like (*J. regia*)	–2.37
miR397b	2.05	TRINITY_DN26934_c1_g1	Laccase-17-like (*J. regia*)	–1.83
miR397b	2.05	TRINITY_DN27015_c0_g1	Laccase-17-like (*J. regia*)	–1.54
miR397b	2.05	TRINITY_DN28629_c3_g4	Laccase-7-like (*J. regia*)	–2.37
miR397c	1.86	TRINITY_DN27015_c0_g1	Laccase-17-like (*J. regia*)	–1.54
miR397c	1.86	TRINITY_DN26934_c1_g1	Laccase-17-like (*J. regia*)	–1.83
miR397c	1.86	TRINITY_DN28629_c3_g4	Laccase-7-like (*J. regia*)	–2.37
miR403a	1.50	TRINITY_DN27428_c1_g4	*AGO2* (*J. regia*)	–1.05

*-inf^a^ indicates that no read was detected in LP_R or LP_SL sample, while the counts were observed in CK_R or CK_SL sample; inf^*b*^ indicates that no read was detected in CK_R sample, while the counts were observed in LP_R sample. The English in this document has been checked by at least two professional editors, both native speakers of English. For a certificate, please see: http://www.textcheck.com/certificate/Mh4hCj.*

In shoots, 112 pairs involving 38 DE miRNAs and 87 target genes were subjected to further analysis. Thus, 23 pairs including 13 DE miRNAs and 16 DE mRNAs were identified, of which 16 miRNA–mRNA pairs showed a negative correlation under Pi starvation (Table 2). For example, miR159a was significantly repressed with log_2_FC of −1.18, and the corresponding target gene GAMYB was significantly induced with log_2_FC of 1.25 under LP conditions. Three miR397 members were upregulated, and four *Laccase* genes were significantly repressed. Unexpectedly, only blu-miR40-5′ and miR319b-5′ were common to roots and shoots, and were downregulated under LP conditions, whereas the corresponding target E3 ubiquitin-protein ligase MIEL1-like gene and one putative mitochondrial protein gene were induced. KEGG analysis showed that E3 ubiquitin-protein ligase MIEL1-like gene participated in “ubiquitin mediated proteolysis” (ko04120).

**FIGURE 6:**
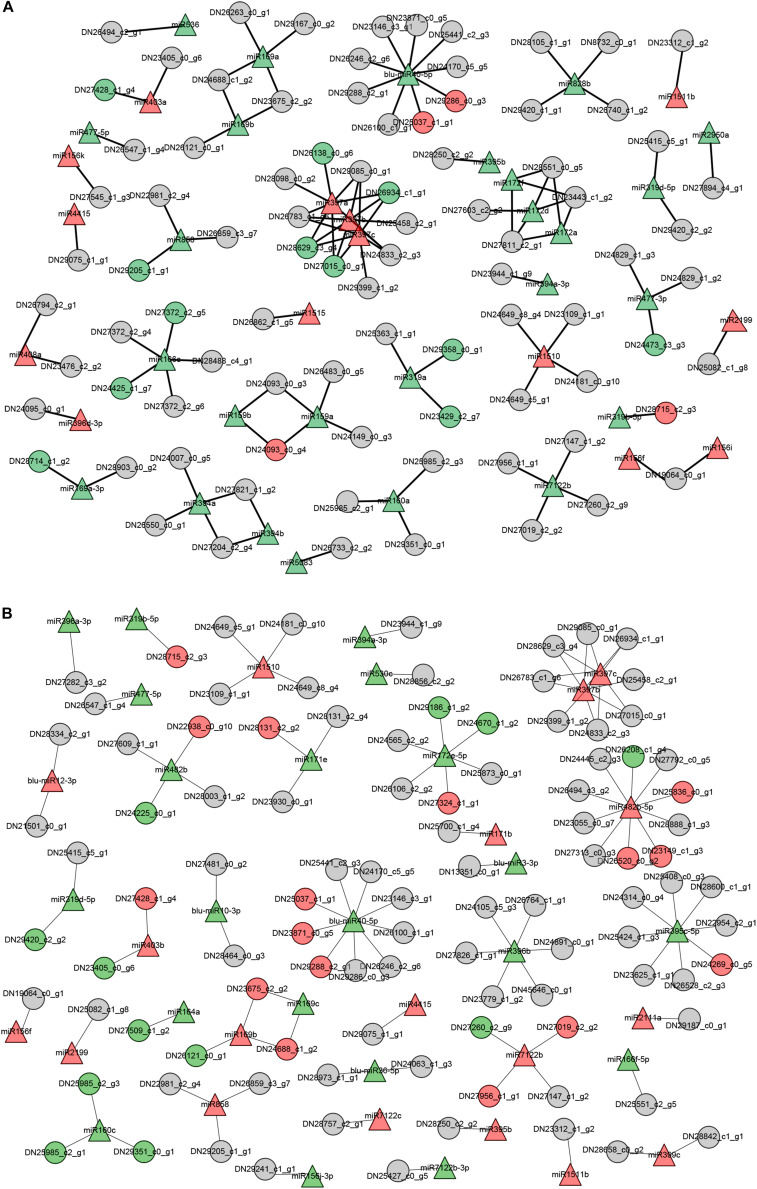
The putative functional networks for –Pi-responsive miRNAs and their corresponding target genes in *B*. *luminifera*. The network depicts the relationship of –Pi-responsive miRNAs and target genes from their expression patterns upon Pi deficiency in shoots **(A)** and roots **(B)**. Triangle indicates miRNAs and circles are target genes. Red represents upregulated, green indicates downregulated, and gray means unchanged under Pi starvation.

As summarized in Figure 6, the shoots and roots of *B. luminifera* showed unique expression profiles of miRNA-target pairs when subjected to LP conditions. These observations suggest that miRNAs may show distinct responses to Pi starvation in different tissues/organs. Of these miRNA-target pairs, three miR397 members showed negative relationship with four *Laccase* genes in shoots, while miR169c was showed negative correlation with two *NFYA* genes in roots, and one novel miRNA blu-miR40-5p regulated distinct target genes in roots and shoots, respectively. However, some miRNAs showed similar expression patterns with corresponding target genes. For example, miR319a and two targets were inhibited in shoots, and miR160c and three targets were downregulated in roots under Pi starvation.

### Expression Patterns of MiRNAs and Their Target Genes Under Conditions of Pi Deficiency

**FIGURE 7:**
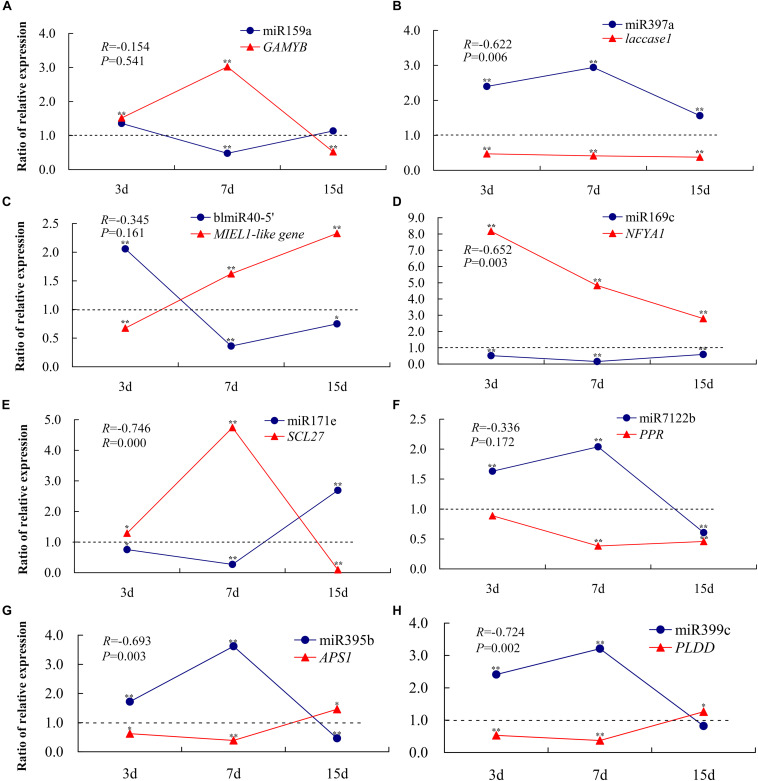
qRT-PCR-derived expression analyses of eight miRNAs and their target genes under –Pi stress. To show the expression patterns of miRNAs and their corresponding target genes more directly, the ratio (K value) is used in the figure instead of individual relative expression levels. K is the ratio of the relative expression levels of LP (–Pi treatment) and CK (normal treatment); i.e., *K* = LP/CK. Each experimental time has a corresponding control group. A *K*-value > 1 indicates upregulated expression compared to the control, while a *K*-value < 1 indicates downregulated expression compared to the control. *P < 0.05; **P < 0.01. The expression levels of miR159a-*GAMYB* **(A)**, miR397a-*laccase1* **(B)**, and BlmiR40-5′-*MIEL1-like* **(C)** were detected in the shoots, and miR169c-*NFYA1* **(D)**, miR171e*-SCL27* **(E)**, miR7122b-*PPR* **(F)**, miR395b-*APS1* **(G)**, and miR399c-*PLDD* **(H)** in the roots were measured. All expression levels were normalized to *TUA* and *MDH* in shoots and roots, respectively. The experiments were repeated three times. Error bars indicate standard deviation.

To confirm the RNA-Seq data and the dynamic correlation between miRNAs and their target genes under conditions of Pi deficiency, the expression changes in eight selected miRNAs and their corresponding target genes after 3, 7, and 15 days of Pi deficiency were assayed using qRT-PCR (Figure 7). The trends in expression patterns of the eight miRNAs and target genes obtained were similar between qRT-PCR and RNA-Seq, and an overall negative correlation between the expression of the miRNAs and their corresponding targets was observed. Specifically, in the shoots, miR159a was induced non-significantly after 3 days, and was then downregulated significantly after 7 days, but was again non-significantly induced after 15 days of Pi deficiency. The target *GAMYB* was upregulated after 3 and 7 days, and then downregulated after 15 days. miR397a was induced significantly under LP condition, and the corresponding target *laccase1* was significantly downregulated, showing a significant negative correlation. BlmiR40-5′ was upregulated after 3 days, and then inhibited after 7 and 15 days of Pi deficiency, and the target *MIEL1-like* showed a negative expression pattern. In the roots, miR169c was inhibited under LP condition, and *NFYA1* was significantly upregulated, showing a significant negative correlation; miR171e was downregulated after 3 and 7 days of Pi deficiency, while the expression level was induced significantly after 15 days of Pi deficiency, and the target *SCL27* showed the opposite expression pattern; three miRNAs including miR7122b, miR395b, and miR399c showed similar expression pattern, exemplified by they were upregulated after 3 and 7 days of Pi deficiency, but were inhibited after 15 days, whereas the corresponding target *PPR*, *APS1*, and *PLDD* were downregulated after 3 and 7 days of LP treatment, of which miR395b and miR399c were significantly and negatively correlated with their target genes.

**FIGURE 8:**
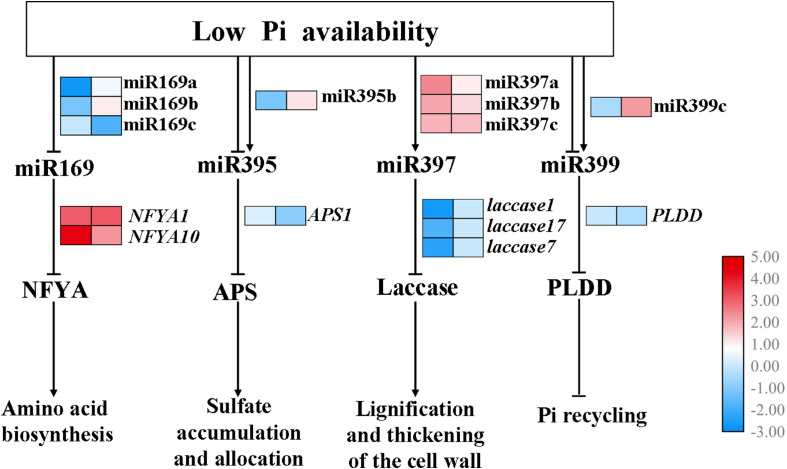
Hypothetical model for the functions of miR169, miR395, miR397, and miR399 in response to Pi starvation. miR169a/b/c, miR395b, miR397a/b/c, and miR399c showed common and unique expression profiles in the shoots and roots when subjected to low Pi availability, then specifically inhibited expression of various target genes, thus affecting downstream biological processes. Arrows and blunted lines indicate positive and negative interactions, respectively. The left and right columns represent the log2(LP/CK) of the transcriptional levels in shoots and roots, respectively.

Furthermore, a putative model has been proposed (Figure 8). Several miRNA-target pairs showed common and unique expression profiles in the shoots and roots, when the seedlings of *B. luminifera* encounter the Pi deficiency stress. Specifically, the expression of miR169a/b and miR169c were inhibited in shoots and roots, respectively, while their corresponding target *NFYA1/10* were induced; miR395b was downregulated and was induced in shoots and roots, respectively, while the target *APS1* showed negative relationship in roots; miR397a/b/c were upregulated, and their targets *Laccase 1/7/17* were inhibited in shoots; miR399c was induced in roots, and target *PLDD* was downregulated upon Pi deficiency stress.

## Discussion

Plants, particularly forestry species, frequently face LP availability in soils and have thus evolved a series of responses and adaptive mechanisms to cope with conditions of Pi deficiency. Understanding the underlying molecular mechanisms is important for selecting and/or producing P-efficient cultivars for modern forestry. The miRNA-mediated posttranscriptional regulation of plant resistance to nutritional deficiency has been described in several plants. In contrast, the molecular mechanisms underlying the responses of forestry trees to Pi deficiency and the posttranscriptional regulation triggered by miRNAs are poorly understood. Based on miRNA, degradome sequencing, and RNA-Seq analyses, we investigated the regulatory roles of miRNAs in roots and shoots of *B. luminifera* under conditions of Pi deficiency. Our results indicated that the genes involved in miRNA-mediated posttranscriptional regulation play an important role in the root and shoot responses of *B*. *luminifera* to Pi deficiency stress. However, our findings provide only initial insights into this topic, and a great deal of further effort will be required to gain a full understanding of the posttranscriptional regulation related to Pi deficiency.

### sRNA Length Patterns and Potential Roles in *Betula luminifera*

Many annual herbaceous plants, such as *A. thaliana* and *Oryza sativa*, contain substantially more 24-nt sRNAs than 21-nt sRNAs (Jones-Rhoades et al., 2006; Morin et al., 2008a), whereas the major peak of redundant sRNAs occurs at 21 nt in woody plants, such as Chinese white poplar (Chen et al., 2012), and *B. luminifera* (Zhang J.H. et al., 2016). However, further studies have shown that sRNA compositions are dependent on developmental stages, organs, and/or external stressors (Slotkin et al., 2009; Zhang et al., 2013; Pan et al., 2017). We found that the length distribution pattern of sRNAs in roots differed significantly from that of shoots, as exemplified by the major peak of redundant sequences at 20 nt in CK roots, in contrast to the major peak at 18 nt and minor peak at 20 nt in LP roots (Figure 3A). Pei et al. (2013) suggested that the abundance of sRNAs may be related to Pi tolerance, as exemplified by the lower relative abundances of 21-nt and 24-nt sRNAs in the LP-tolerant mutant 99038 compared to wild-type Qi319 maize under LP conditions. Further analysis of the components of sRNAs with lengths 18 nt and 19 nt revealed that a large proportion of 18–20-nt sequences originated from tRNAs. The tRNA ratios with sizes of 18 and 19 nt were significantly increased in LP-treated roots, especially for 18 nt, whereas the corresponding ratios were significantly decreased in LP-treated shoots (Supplementary Table 4). Previous studies revealed the accumulation of tRNA fragments in roots of P-starved *Arabidopsis* (Hsieh et al., 2009) and in nitrate-starved seedlings (Liang et al., 2012). Hsieh et al. (2009) reported that the 19-nt RNAs from Gly-tRNA^*TCC*^ and Asp-tRNA^*GTC*^ corresponding to the 5′ end of tRNA were highly accumulated in roots but were present at much lower levels in shoots, indicating a spatial and temporal expression pattern of sRNAs derived from specific cleavage of tRNAs rather than random degradation. Several studies have indicated that tRNA-derived sRNAs accumulate in specific tissues or under specific stress conditions (Thompson et al., 2008; Fu et al., 2009). Furthermore, Zhang et al. (2009) reported that tRNA halves detected in phloem sap of pumpkin (*Cucurbita maxima*) inhibited translational activity *in vitro*, suggesting that these tRNA halves may act as long-distance signals to coordinate the metabolic status between source and sink tissues. Recent studies have shown that tRNA fragments are incorporated into Argonaute (AGO) complexes, and are likely to regulate gene expression post-transcriptionally in a manner similar to miRNAs (Loss-Morais et al., 2013).

Apart from tRNA fragments, the isomiR accumulation was observed in our study. It exemplified by several isomiRs with a length bias of 18 or 19 nt were only differentially expressed in roots and had more diversified targets, such as two targets for miR399c, eight targets for miR395c-5′, and 10 targets for miR482-5′ (Supplementary Table 9). Recent evidence suggests that isomiR production is differentially modulated at different developmental stages and by adverse environmental signals (Hackenberg et al., 2013; Balyan et al., 2020; Fard et al., 2020). For example, Shao et al. (2015) found that the abundance of iso-osa-miR528-5p in AGO1 complexes was higher than that of its canonical osa-miR528-5p in rice. The 19 nt isomiR of pvu-miR171a was exclusively detected in flowers, whereas the 20 nt isomiR of pvu-miR479 was highly expressed in roots (Pelaez et al., 2012). The isomiRs that are shorter than their canonical miRNAs could have been subjected to nucleotide truncation by exonucleolytic digestion (Lu et al., 2009), or they could simply have been synthesized to a shorter length compared with their dominant miRNAs (Morin et al., 2008b). The trimming of the 3′ end shortens the length of canonical miRNAs and increases their stability in AGO protein complexes (Ameres et al., 2010). Subtle differences in miRNA sequences can affect their AGO sorting, even for isomiRs derived from the same miRNA precursor (Iki et al., 2018); the alteration in the lengths of isomiRs could therefore be a cellular strategy to diversify miRNA target specificity by differential loading into AGO complexes. On the other hand, different isomiRs may regulate specific targets. For example, Naya et al. (2010) showed that the 20 nt isomiR of miR156 cleaved a new WD40-like protein target. Therefore, the diversified target genes triggered by the isomiR accumulation might be an important mechanism in response to Pi deficiency in *B*. *luminifera.*

### Divergent Expression of MiRNAs Between Roots and Shoots Under Conditions of Pi Deficiency

There is accumulating evidence that miRNA-mediated genes are involved in basic metabolic processes, root and shoot development, stress responses, and Pi uptake (Li et al., 2018). However, many studies have reported that changes in the expression of genes, including *MIRNA* genes, were organ-dependent (Aceituno et al., 2008; Xu et al., 2013). Aceituno et al. (2008) reported that 10-fold more genes were differentially expressed between organs as compared to any other experimental variable. Xu et al. (2013) showed that a series of miRNAs showed leaf- or root-specific expression under Pi-sufficient and -deficient conditions in soybean. Similar phenomena were observed in cucumber, with some miRNAs preferentially expressed in certain tissues (Mao et al., 2012). In the present study, 43 and 37 miRNAs were specifically differentially expressed in roots and shoots, respectively (Supplementary Figure 3). Therefore, the divergent expressions of miRNAs were detected among different organs in response to Pi deficiency. Further GO enrichment analysis of targets for DE miRNAs showed that –Pi responsive miRNA-target pairs were mainly classified into transcription regulation, oxidoreductase activity, ATP binding, copper ion binding, defense response to bacterium, lignin biosynthetic process, and leaf development categories. It suggested that these miRNA-target pairs trigger removing of H_2_O_2_ because of Pi stress, activating defense response, and regulating growth. Similar phenomenon was observed in maize, which is exemplified by miRNA-target pairs alter the morphology, physiology or metabolism of plants upon prolonged Pi deficiency (Li et al., 2016).

The putative functional networks of miRNA-target pairs presented that miRNAs show distinct responses to Pi starvation in different tissues/organs (Figure 6). A large proportion of DE miRNAs showed stress-related expression. For example, miR397 and miR398 were upregulated in both roots and shoots of Pi-starved *B*. *luminifera*. This is in contrast to studies in soybean roots where miR397a was induced by −Pi conditions in soybean roots, whereas miR398a, miR398b, miR398c, and miR408 were repressed in Pi-depleted leaves (Xu et al., 2013); miR397a, miR397b, miR398a, and miR398b were co-downregulated in both the leaves and roots of maize (Li et al., 2016), indicating divergent roles for these stress-related miRNA families between plant tissues and/or species. In *B*. *luminifera*, three miR397 members showed significant negative correlations with four *laccase* genes under −Pi conditions in shoots, but not in roots (Table 2). Previous studies have shown that *laccase* genes are related to lignification and thickening of the cell wall in secondary cell growth (Constabel et al., 2000). Khandal et al. (2020) aslo showed a regulatory role for the miR397b-*LAC2* module in root lignification during Pi deficiency. Therefore, we speculate that miR397 may mainly regulate *laccase* genes in shoots, and increased miR397 expression may contribute to the decreased *laccase* mRNA levels, participating in the shoot lignification and thickening of cell wall under −Pi conditions in *B*. *luminifera.*

miR169 members showed differential changes in expression in roots and shoots of maize under LP conditions, as four miR169 members (miR169f/g/h/l) were upregulated in leaves but miR169a was repressed only in roots after 4 days of treatment (Li et al., 2016). In the present study, miR169b was induced and miR169c was repressed in roots, whereas miR169a/b was inhibited in shoots. Similarly, soybean miR169c/r were repressed both in leaves and roots, while miR169q was upregulated in leaves under LP conditions (Xu et al., 2013). Previous studies showed miR169-*NYFAs* participate in nitrogen and drought stress responses (Zhao et al., 2011). In *B. luminifera*, miR169c was downregulated in −Pi-treated roots, and the corresponding targets *NFYA1* and *NFYA10* were significantly induced (Table 2). *NFYA10* was predicted to participate in aminoacyl-tRNA biosynthesis. Previous studies have shown that aminoacyl-tRNA synthetases (aaRSs) ensure the fidelity of translation of the genetic code, covalently attaching appropriate amino acids to the corresponding nucleic acid adaptor molecule–tRNA (Klipcan and Safro, 2004). The aminoacylation reaction catalyzed by aaRSs is the most fundamental step for amino acid biosynthesis. In addition, Pant et al. (2008) indicated that miR169 is a potential long-distance signal that can report shoot Pi status to the roots, similar to the role of miR399. Therefore, miR169-*NFYAs* might have a multifunctional role in response to Pi starvation in *B*. *luminifera*.

Previous studies showed that miR395 was induced by low sulfate treatment, involved in the regulation of sulfate accumulation and allocation by targeting *APS* genes (Liang et al., 2010). miR395b was specifically induced in −Pi-treated roots, and the target *APS1* was downregulated in *B. luminifera* (Figure 7). Several studies have indicated that miR395 and miR399 are transported from shoots to roots *via* the phloem (Buhtz et al., 2010), which it was consistent with the decreasing level of miR395b in *B. luminifera* shoots. Therefore, miR395 may act as a mobile signal and travel long distances to induce systemic silencing of *APS* and other targets, thus contributing to adaptation to −Pi environments in *B. luminifera*.

miR399 is responsive to Pi starvation, which demonstrates its important regulatory role in Pi homeostasis (Fujii et al., 2005; Chiou et al., 2006; Pant et al., 2008). Four miR399 members were identified in our library, which cleave *PHO2* mRNA based on our degradome data. However, only miR399c, a 19 nt isomiR that contains two truncated nucleotides at the 3′ end of canonical miR399a, was induced significantly in LP-treated *B. luminifera* roots. A similar phenomenon was observed in barley (*H. vulgare*), in which several isomiRs of miR399 and miR827 were also significantly upregulated under conditions of Pi starvation (Hackenberg et al., 2013). In our study, *PLDD* was firstly found to be a cleavage target of miR399c. *PLDD*, which belongs to the *PLD* family, was inhibited in a manner typical for PLD family proteins. PLD is involved in multiple plant processes, ranging from abiotic and biotic stress responses to plant development. *PLDD-*knockout mutants are tolerant to severe drought stress, indicating that *PLDD* acts as a negative regulator in response to drought conditions (Distefano et al., 2015). This suggests that *PLDD* might play a negative regulatory role in *B*. *luminifera* under LP conditions. Therefore, the induction of isomiRs, such as miR399c, in LP-treated roots of *B*. *luminifera* to reduce the *PLDD* abundance, which may improve the tolerance to Pi starvation.

miR827-*NLA* pair was reported to be involved in regulating Pi homeostasis (Hsieh et al., 2009; Kant et al., 2011). However, miR827 was absent in both of present sRNA libraries and our previous analyses of *B*. *luminifera* (Zhang J.H. et al., 2016), except for five reads detected after 4 h of heat stress (Pan et al., 2017). Because of the lack of its precursor sequence in the draft genome, the presence of miR827 in *B*. *luminifera* is inconclusive. Its absence could be explained either by loss of *MIR827* during evolution or by failed ligation during sRNA library construction. Similar phenomena were observed in three legumes and *C*. *papaya*, in which the *MIR827* locus was lost during evolution (Lin et al., 2018).

## Conclusion

Integrated analysis of mRNA-Seq, miRNA-Seq, and degradome-Seq was performed to elucidate the molecular mechanisms underlying the response of *B. luminifera* to conditions of Pi deficiency. Totals of 8,095 and 5,584 DE mRNAs were identified in –Pi-treated roots and shoots, respectively. sRNA sequencing showed that 66 and 60 miRNAs were identified as −Pi-responsive miRNAs, and 109 and 112 miRNA–target pairs were further validated in roots and shoots, respectively, thus a network was proposed. Taken together, these findings provide useful information to decipher miRNA functions and establish a framework for exploring P signaling networks regulated by miRNAs in *B. luminifera* and other woody plants.

## Data Availability Statement

We have uploaded our raw data to the NCBI and the accession number of transcriptome data were GSM4105637–GSM4105640, and the accession number of sRNA data were GSM4105633–GSM4105636.

## Author Contributions

JZ and ZT conceived and designed the experiments. JZ, YL, and LC analyzed the high-throughput sequencing data. YL, FW, and MH contributed reagents and materials. YZ, YL, and LC performed the qRT-PCR experiment. JZ and ZT wrote and revised the manuscript. All authors read and approved the final manuscript.

## Conflict of Interest

The authors declare that the research was conducted in the absence of any commercial or financial relationships that could be construed as a potential conflict of interest.

## Footnotes

1 http://www.mirbase.org

2 http://bioinfo.cau.edu.cn/agriGO/

## Supplementary Material

Supplementary Figure 1 KEGG pathway enrichment analysis of DE mRNAs under −Pi stress. The 20 most-enriched KEGG pathways among the DE mRNAs identified in **(A)** roots and **(B)** shoots under −Pi stress using KOBAS (2.0) with FDR ≤ 0.05.

Supplementary Figure 2 Validation of the expression profiles of genes in *B. luminifera* shoots and roots identified by RNA-seq using qRT-PCR. **(A)** Relative expression of eight LP-responsive genes in shoots after 3, 7, and 15 days of LP treatment; **(B)** Relative expression of eight LP-responsive genes in roots after 3, 7, and 15 days of LP treatment. Expression level is represented by the ratio of LP treatment to CK. The value of relative expression level above one indicates that genes are upregulated by Pi starvation, and the value below one shows that genes are downregulated by Pi starvation. All expression levels were normalized to *TUA* and *MDH* in shoots and roots, respectively. The experiments were repeated three times. Error bars indicate standard deviation.

Supplementary Figure 3 Identification of DE miRNAs in shoots and roots after 7 days of Pi starvation. DE miRNAs in response to 7 days of Pi starvation in **(A)** shoots and **(B)** roots were identified using Fisher’s exact test, with the significance threshold set to 0.05. Only significantly differentially expressed miRNAs with a fold change greater than 2 are shown. **(C)** Venn diagrams showing the numbers of common DE miRNAs and the overlapping sets obtained across four comparisons.

Supplementary Figure 4 Validation of miRNA target genes. Target plots (t-plots) of representative miRNA targets confirmed by degradome-Seq. The abundance of each signature is plotted as a function of its position in the transcript. The representative miRNAs and their corresponding targets are shown; the red line indicates the cleavage site of each transcript. *ARF17*, auxin response factor 17; *NAC21*, (NAM, ATAF, CUC 21); *NFYA1*, nuclear transcription factor Y subunit A1; *APS1*, ATP sulfurylase 1; *MYB108*, Myb domain protein 108.

Supplementary Figure 5 Possible functional networks for −Pi-responsive miRNAs and their corresponding target genes in *B*. *luminifera*. Relationships between −Pi-responsive miRNAs and their target genes shown based upon putative physiological functions. Red arrows represent upregulation, whereas green arrows represent downregulation.

Supplementary Table 1 5′RLM-RACE primers for validation of miRNA targets.

Supplementary Table 2 Gene primers used in qRT-PCR.

Supplementary Table 3 miRNA primers used in qRT-PCR.

Supplementary Table 4 tRNA identification in sRNA libraries.

Supplementary Table 5 Identification of known miRNAs in *B. luminifera*.

Supplementary Table 6 Identification of novel miRNAs in *B. luminifera*.

Supplementary Table 7 Differences in expression of miRNAs between control and low phosphate (LP) treatment in roots.

Supplementary Table 8 Differences in expression of miRNAs between control and LP treatment in shoots.

Supplementary Table 9 miRNA targets in roots.

Supplementary Table 10 miRNA targets in shoots.

Supplementary Table 11 Gene ontology (GO) enrichment analysis for targets of DE miRNAs under conditions of phosphate (Pi) starvation.

Supplementary Table 12 Kyoto Encyclopedia of Genes and Genomes (KEGG) enrichment analysis for targets of DE miRNAs under conditions of Pi starvation.

## References

[B1] Aceituno F. F. Moseyko N. Rhee S. Y. Gutierrez R. A. (2008). The rules of gene expression in plants: organ identity and gene body methylation are key factors for regulation of gene expression in *Arabidopsis thaliana*. *BMC Genom.* 9:438. 10.1186/1471-2164-9-438 18811951 PMC2566314

[B2] Ameres S. L. Horwich M. D. Hung J. H. Xu J. Ghildiyal M. Weng Z. (2010). Target RNA-directed trimming and tailing of small silencing RNAs. *Science* 328 1534–1539. 20558712 10.1126/science.1187058 PMC2902985

[B3] Bai Q. Q. Wang X. Y. Chen X. Shi G. Q. Liu Z. P. Guo C. J. (2018). Wheat miRNA TaemiR408 acts as an essential mediator in plant tolerance to Pi deprivation and salt stress via modulating stress-associated physiological processes. *Front. Plant Sci.* 9:499. 10.3389/fpls.2018.00499 29720988 PMC5916090

[B4] Balyan S. Joseph S. V. Jain R. Mutum R. D. Functional S. R. J. Genomics I. (2020). Investigation into the miRNA/5 ’ isomiRNAs function and drought-mediated miRNA processing in rice. *Funct. Integr. Genom.* 20 509–522. 10.1007/s10142-020-00731-2 31925598

[B5] Buhtz A. Pieritz J. Springer F. Kehr J. (2010). Phloem small RNAs, nutrient stress responses, and systemic mobility. *BMC Plant Biol.* 10:64. 10.1186/1471-2229-10-64 20388194 PMC2923538

[B6] Charles Addo-Quaye T. Eshoo W. Bartel D. P. Michael J. A. (2008). Endogenous siRNA and miRNA targets identified by sequencing of the *Arabidopsis* degradome. *Curr. Biol.* 18 758–762. 10.1016/j.cub.2008.04.042 18472421 PMC2583427

[B7] Chen L. Ren Y. Zhang Y. Xu J. Sun F. Zhang Z. (2012). Genome-wide identification and expression analysis of heat-responsive and novel microRNAs in *Populus tomentosa*. *Gene* 504 160–165. 10.1016/j.gene.2012.05.034 22634103

[B8] Chen Y. F. Li L. Q. Xu Q. Kong Y. H. Wang H. Wu W. H. (2009). The WRKY6 transcription factor modulates PHOSPHATE1 expression in response to low Pi stress in *Arabidopsis*. *Plant Cell* 21 3554–3566. 10.1105/tpc.108.064980 19934380 PMC2798333

[B9] Chiou T. J. Aung K. Lin S. I. Wu C. C. Chiang S. F. Su C. L. (2006). Regulation of phosphate homeostasis by microRNA in *Arabidopsis*. *Plant Cell* 18 412–421. 10.1105/tpc.105.038943 16387831 PMC1356548

[B10] Constabel C. P. Yip L. Patton J. J. Christopher M. E. (2000). Polyphenol oxidase from hybrid poplar. Cloning and expression in response to wounding and herbivory. *Plant Physiol.* 124 285–295. 10.1104/pp.124.1.285 10982443 PMC59143

[B11] Cordell D. Drangert J.-O. White S. (2009). The story of phosphorus: global food security and food for thought. *Glob. Environ. Chang.* 19 292–305. 10.1016/j.gloenvcha.2008.10.009

[B12] Distefano A. M. Valinas M. A. Scuffi D. Lamattina L. Ten Have A. Garcia-Mata C. (2015). Phospholipase D delta knock-out mutants are tolerant to severe drought stress. *Plant Signal. Behav.* 10:e1089371. 10.1080/15592324.2015.1089371 26340512 PMC4883880

[B13] Du Z. Zhou X. Ling Y. Zhang Z. Su Z. (2010). agriGO: a GO analysis toolkit for the agricultural community. *Nucleic Acids Res.* 38 W64–W70. 20435677 10.1093/nar/gkq310 PMC2896167

[B14] Duff S. M. G. Sarath G. Plaxton W. C. (1994). The role of acid phosphatases in plant phosphorus metabolism. *Physiol. Plant.* 90 791–800. 10.1111/j.1399-3054.1994.tb02539.x

[B15] Fard E. M. Moradi S. Salekdeh N. N. Bakhshi B. Ghaffari M. R. Zeinalabedini M. (2020). Plant isomiRs: origins, biogenesis, and biological functions. *Genomics* 112 3382–3395. 10.1016/j.ygeno.2020.06.019 32561347

[B16] Fu H. Feng J. Liu Q. Sun F. Tie Y. Zhu J. (2009). Stress induces tRNA cleavage by angiogenin in mammalian cells. *FEBS Lett.* 583 437–442. 10.1016/j.febslet.2008.12.043 19114040

[B17] Fujii H. Chiou T. J. Lin S. I. Aung K. Zhu J. K. (2005). A miRNA involved in phosphate-starvation response in *Arabidopsis*. *Curr. Biol.* 15 2038–2043. 10.1016/j.cub.2005.10.016 16303564

[B18] Hackenberg M. Shi B. J. Gustafson P. Langridge P. (2013). Characterization of phosphorus-regulated miR399 and miR827 and their isomirs in barley under phosphorus-sufficient and phosphorus-deficient conditions. *BMC Plant Biol.* 13:214. 10.1186/1471-2229-13-214 24330740 PMC3878733

[B19] Hamburger D. Rezzonico E. Macdonald-Comber Petetot J. Somerville C. Poirier Y. (2002). Identification and characterization of the *Arabidopsis* PHO1 gene involved in phosphate loading to the xylem. *Plant Cell* 14 889–902. 10.1105/tpc.000745 11971143 PMC150690

[B20] Hsieh L. C. Lin S. I. Shih A. C. Chen J. W. Lin W. Y. Tseng C. Y. (2009). Uncovering small RNA-mediated responses to phosphate deficiency in *Arabidopsis* by deep sequencing. *Plant Physiol* 151 2120–2132. 10.1104/pp.109.147280 19854858 PMC2785986

[B21] Huang H. H. Jiang C. Tong Z. K. Cheng L. J. Zhu M. Y. Lin E. P. (2014). Eight distinct cellulose synthase catalytic subunit genes from *Betula luminifera* are associated with primary and secondary cell wall biosynthesis. *Cellulose* 21 2183–2198. 10.1007/s10570-014-0261-z

[B22] Huang J. Huang Z. Zhou X. Xia C. Imran M. Wang S. (2019). Tissue-specific transcriptomic profiling of Plantago major provides insights for the involvement of vasculature in phosphate deficiency responses. *Mol. Genet. Genom.* 294 159–175. 10.1007/s00438-018-1496-4 30267144

[B23] Iki T. Clery A. Bologna N. G. Sarazin A. Brosnan C. A. Pumplin N. (2018). Structural flexibility enables alternative maturation, ARGONAUTE sorting and activities of miR168, a global gene silencing regulator in plants. *Mol. Plant* 11 1008–1023. 10.1016/j.molp.2018.05.006 29803952

[B24] Jain A. Poling M. D. Smith A. P. Nagarajan V. K. Lahner B. Meagher R. B. (2009). Variations in the composition of gelling agents affect morphophysiological and molecular responses to deficiencies of phosphate and other nutrients. *Plant Physiol.* 150 1033–1049. 10.1104/pp.109.136184 19386810 PMC2689959

[B25] Jones-Rhoades M. W. Bartel D. P. (2004). Computational identification of plant MicroRNAs and their targets, including a stress-induced miRNA. *Mol. Cell* 14 787–799. 10.1016/j.molcel.2004.05.027 15200956

[B26] Jones-Rhoades M. W. Bartel D. P. Bartel B. (2006). MicroRNAs and their regulatory roles in plants. *Annu. Rev. Plant Biol.* 57 19–53. 10.1146/annurev.arplant.57.032905.105218 16669754

[B27] Kant S. Peng M. Rothstein S. J. (2011). Genetic regulation by NLA and microRNA827 for maintaining nitrate-dependent phosphate homeostasis in *Arabidopsis*. *PLoS Genet.* 7:e1002021. 10.1371/journal.pgen.1002021 21455488 PMC3063762

[B28] Khandal H. Singh A. P. Chattopadhyay D. J. P. P. (2020). MicroRNA397b-LACCASE2 module regulates root lignification under water- and phosphate deficiency. *Plant Physiol.* 182:921. 10.1104/pp.19.00921 PMC7054887 31949029

[B29] Klipcan L. Safro M. (2004). Amino acid biogenesis, evolution of the genetic code and aminoacyl-tRNA synthetases. *J. Theor. Biol.* 228 389–396. 10.1016/j.jtbi.2004.01.014 15135037

[B30] Kozomara A. Griffiths-Jones S. (2013). miRBase: annotating high confidence microRNAs using deep sequencing data. *Nucleic Acids Res.* 2013:gkt1181. 10.1093/nar/gkt1181 PMC3965103 24275495

[B31] Kumar S. Verma S. Trivedi P. K. (2017). Involvement of small RNAs in phosphorus and sulfur sensing, signaling and stress: current update. *Front. Plant Sci.* 8:285. 10.3389/fpls.2017.00285 28344582 PMC5344913

[B32] Lei M. Liu Y. Zhang B. Zhao Y. Wang X. Zhou Y. (2011). Genetic and genomic evidence that sucrose is a global regulator of plant responses to phosphate starvation in *Arabidopsis*. *Plant Physiol.* 156 1116–1130. 10.1104/pp.110.171736 21346170 PMC3135933

[B33] Li M. Xia Y. Gu Y. Zhang K. Lang Q. Chen L. (2010). MicroRNAome of porcine pre- and postnatal development. *PLoS One* 5:e11541 10.1371/journal.pgen.0011541 PMC2902522 20634961

[B34] Li Z. Xu H. Li Y. Wan X. Ma Z. Cao J. (2018). Analysis of physiological and miRNA responses to Pi deficiency in alfalfa (*Medicago sativa* L.). *Plant Mol. Biol.* 96 473–492. 10.1007/s11103-018-0711-3 29532290

[B35] Li Z. Zhang X. Liu X. Zhao Y. Wang B. Zhang J. (2016). miRNA alterations are important mechanism in maize adaptations to low-phosphate environments. *Plant Sci.* 252 103–117. 10.1016/j.plantsci.2016.07.009 27717445

[B36] Liang G. Ai Q. Yu D. Q. (2015). Uncovering miRNAs involved in crosstalk between nutrient deficiencies in *Arabidopsis*. *Sci. Rep.* 5:11813. 10.1038/srep11813 PMC4488870 26134148

[B37] Liang G. He H. Yu D. (2012). Identification of nitrogen starvation-responsive microRNAs in *Arabidopsis thaliana*. *PLoS One* 7:e48951. 10.1371/journal.pone.0048951 23155433 PMC3498362

[B38] Liang G. Yang F. Yu D. (2010). MicroRNA395 mediates regulation of sulfate accumulation and allocation in *Arabidopsis thaliana*. *Plant J.* 62 1046–1057. 20374528 10.1111/j.1365-313X.2010.04216.x

[B39] Lin W. Y. Lin S. I. Chiou T. J. (2009). Molecular regulators of phosphate homeostasis in plants. *J. Exper. Bot.* 60 1427–1438. 10.1093/jxb/ern303 19168668

[B40] Lin W. Y. Lin Y. Y. Chiang S. F. Syu C. Hsieh L. C. Chiou T. J. (2018). Evolution of microRNA827 targeting in the plant kingdom. *New Phytol.* 217 1712–1725. 10.1111/nph.14938 29214636

[B41] Liu Y. Mi G. Chen F. Zhang J. Zhang F. (2004). Rhizosphere effect and root growth of two maize (*Zea mays* L.) genotypes with contrasting P efficiency at low P availability. *Plant Sci.* 167 217–223. 10.1016/j.plantsci.2004.02.026

[B42] Loss-Morais G. Waterhouse P. M. Margis R. (2013). Description of plant tRNA-derived RNA fragments (tRFs) associated with argonaute and identification of their putative targets. *Biol. Direct.* 8:6. 10.1186/1745-6150-8-6 PMC3574835 23402430

[B43] Lu S. Sun Y. H. Chiang V. L. (2009). Adenylation of plant miRNAs. *Nucleic Acids Res.* 37 1878–1885. 10.1093/nar/gkp031 19188256 PMC2665221

[B44] Mao W. Li Z. Xia X. Li Y. Yu J. (2012). A combined approach of high-throughput sequencing and degradome analysis reveals tissue specific expression of microRNAs and their targets in cucumber. *PLoS One* 7:e33040 10.1371/journal.pgen.10033040 PMC3316546 22479356

[B45] Mao X. Cai T. Olyarchuk J. G. Wei L. (2005). Automated genome annotation and pathway identification using the KEGG Orthology (KO) as a controlled vocabulary. *Bioinformatics* 21 3787–3793. 10.1093/bioinformatics/bti430 15817693

[B46] Marschner H. (1995). *Mineral Nutrition on Higher Plants.* London: Academic Press.

[B47] Meyers B. C. Axtell M. J. Bartel B. Bartel D. P. Baulcombe D. Bowman J. L. (2008). Criteria for annotation of plant MicroRNAs. *Plant Cell* 20 3186–3190. 10.1105/tpc.108.064311 19074682 PMC2630443

[B48] Morin R. D. Aksay G. Dolgosheina E. Ebhardt H. A. Magrini V. Mardis E. R. (2008a). Comparative analysis of the small RNA transcriptomes of *Pinus contorta* and *Oryza sativa*. *Genome Res.* 18 571–584. 10.1101/gr.6897308 18323537 PMC2279245

[B49] Morin R. D. O’connor M. D. Griffith M. Kuchenbauer F. Delaney A. Prabhu A. L. (2008b). Application of massively parallel sequencing to microRNA profiling and discovery in human embryonic stem cells. *Genome Res.* 18 610–621. 10.1101/gr.7179508 18285502 PMC2279248

[B50] Naya L. Khan G. A. Sorin C. Hartmann C. Crespi M. Lelandais-Briere C. (2010). Cleavage of a non-conserved target by a specific miR156 isoform in root apexes of *Medicago truncatula*. *Plant Signal Behav.* 5, 328–331. 10.4161/psb.5.3.11190 20200496 PMC2881292

[B51] Nilsson L. Muller R. Nielsen T. H. (2010). Dissecting the plant transcriptome and the regulatory responses to phosphate deprivation. *Physiol. Plant* 139 129–143. 10.1111/j.1399-3054.2010.01356.x 20113436

[B52] O’Rourke J. A. Yang S. S. Miller S. S. Bucciarelli B. Liu J. Q. Rydeen A. (2013). An RNA-Seq transcriptome analysis of orthophosphate-deficient white lupin reveals novel insights into phosphorus acclimation in plants. *Plant Physiol.* 161 705–724. 10.1104/pp.112.209254 23197803 PMC3561014

[B53] Pan Y. Niu M. Y. Liang J. S. Lin E. P. Tong Z. K. Zhang J. H. (2017). Identification of heat-responsive miRNAs to reveal the miRNA-mediated regulatory network of heat stress response in *Betula luminifera*. *Trees Struct. Funct.* 31 1635–1652. 10.1007/s00468-017-1575-x

[B54] Pant B. D. Buhtz A. Kehr J. Scheible W. R. (2008). MicroRNA399 is a long-distance signal for the regulation of plant phosphate homeostasis. *Plant J.* 53, 731–738. 10.1111/j.1365-313X.2007.03363.x 17988220 PMC2268993

[B55] Pei L. Jin Z. Li K. Yin H. Wang J. Yang A. (2013). Identification and comparative analysis of low phosphate tolerance-associated microRNAs in two maize genotypes. *Plant Physiol. Biochem.* 70 221–234. 10.1016/j.plaphy.2013.05.043 23792878

[B56] Pelaez P. Trejo M. S. Iniguez L. P. Estrada-Navarrete G. Covarrubias A. A. Reyes J. L. (2012). Identification and characterization of microRNAs in *Phaseolus vulgaris* by high-throughput sequencing. *BMC Genom.* 13:83. 10.1186/1471-2164-13-83 22394504 PMC3359237

[B57] Secco D. Jabnoune M. Walker H. Shou H. Wu P. Poirier Y. (2013). Spatio-temporal transcript profiling of rice roots and shoots in response to phosphate starvation and recovery. *Plant Cell* 25 4285–4304. 10.1105/tpc.113.117325 24249833 PMC3875719

[B58] Shannon P. Markiel A. Ozier O. Baliga N. S. Wang J. T. Ramage D. (2003). Cytoscape: a software environment for integrated models of biomolecular interaction networks. *Genome Res.* 13 2498–2504. 10.1101/gr.1239303 14597658 PMC403769

[B59] Shao H. Wang H. Tang X. (2015). NAC transcription factors in plant multiple abiotic stress responses: progress and prospects. *Front Plant Sci.* 6:902. 10.3389/fpls.2015.00902 26579152 PMC4625045

[B60] Shin H. Shin H. S. Dewbre G. R. Harrison M. J. (2004). Phosphate transport in Arabidopsis: Pht1;1 and Pht1;4 play a major role in phosphate acquisition from both low- and high-phosphate environments. *Plant J.* 39 629–642. 15272879 10.1111/j.1365-313X.2004.02161.x

[B61] Slotkin R. K. Vaughn M. Borges F. Tanurdzic M. Becker J. D. Feijo J. A. (2009). Epigenetic reprogramming and small RNA silencing of transposable elements in pollen. *Cell* 136 461–472. 10.1016/j.cell.2008.12.038 19203581 PMC2661848

[B62] Su T. Xu Q. Zhang F. C. Chen Y. Li L. Q. Wu W. H. (2015). WRKY42 modulates phosphate homeostasis through regulating phosphate translocation and acquisition in *Arabidopsis*. *Plant Physiol.* 167 1579–U1717. 25733771 10.1104/pp.114.253799 PMC4378159

[B63] Sun S. Gu M. Cao Y. Huang X. Zhang X. Ai P. (2012). A constitutive expressed phosphate transporter, OsPht1;1, modulates phosphate uptake and translocation in phosphate-replete rice. *Plant Physiol.* 159 1571–1581. 10.1104/pp.112.196345 22649273 PMC3425197

[B64] Sunkar R. Girke T. Jain P. K. Zhu J. K. (2005). Cloning and characterization of microRNAs from rice. *Plant Cell* 17, 1397–1411. 10.1105/tpc.105.031682 15805478 PMC1091763

[B65] Sunkar R. Li Y. F. Jagadeeswaran G. (2012). Functions of microRNAs in plant stress responses. *Trends Plant Sci.* 17 196–203. 10.1016/j.tplants.2012.01.010 22365280

[B66] Takahashi A. Takeda K. Ohnishi T. (1991). Light-induced anthocyanin reduces the extent of damage to DNA in UV-irradiated *Centaura cyanus* cells in culture. *Plant Cell Physiol.* 32 541–547.

[B67] Thompson D. M. Cheng L. Green P. J. Parker R. (2008). tRNA cleavage is a conserved response to oxidative stress in eukaryotes. *RNA* 14 2095–2103. 10.1261/rna.1232808 18719243 PMC2553748

[B68] Tran H. T. Hurley B. A. Plaxton W. C. (2010). Feeding hungry plants: the role of purple acid phosphatases in phosphate nutrition. *Plant Sci.* 179 14–27. 10.1016/j.plantsci.2010.04.005

[B69] Trapnell C. Roberts A. Goff L. Pertea G. Kim D. Kelley D. R. (2012). Differential gene and transcript expression analysis of RNA-seq experiments with TopHat and Cufflinks. *Nat. Protoc.* 7 562–578. 10.1038/nprot.2012.016 22383036 PMC3334321

[B70] Vance C. P. Uhde-Stone C. Allan D. L. (2003). Phosphorus acquisition and use: critical adaptations by plants for securing a nonrenewable resource. *New Phytol.* 157 427–447. 10.1046/j.1469-8137.2003.00695.x 33873400

[B71] Wege S. Khan G. A. Jung J. Y. Vogiatzaki E. Pradervand S. Aller I. (2016). The EXS domain of PHO1 participates in the response of shoots to phosphate deficiency via a root-to-shoot signal. *Plant Physiol.* 170 385–400. 10.1104/pp.15.00975 26546667 PMC4704572

[B72] Xu F. Liu Q. Chen L. Kuang J. Liao H. (2013). Genome-wide identification of soybean microRNAs and their targets reveals their organ-specificity and responses to phosphate starvation. *BMC Genom.* 14:66. 10.1186/1471-2164-14-66 23368765 PMC3673897

[B73] Xue Y. Zhuang Q. Zhu S. Xiao B. Liang C. Liao H. (2018). Genome wide transcriptome analysis reveals complex regulatory mechanisms underlying phosphate homeostasis in soybean nodules. *Int. J. Mol. Sci.* 19:2924. 10.3390/ijms19102924 30261621 PMC6213598

[B74] Ye Q. Wang H. Su T. Wu W. H. Chen Y. F. (2018). The ubiquitin E3 Ligase PRU1 regulates WRKY6 degradation to modulate phosphate homeostasis in response to low-Pi stress in *Arabidopsis*. *Plant Cell* 30 1062–1076. 10.1105/tpc.17.00845 29567663 PMC6002188

[B75] Young M. D. Wakefield M. J. Smyth G. K. Oshlack A. (2010). Gene ontology analysis for RNA-seq: accounting for selection bias. *Genome Biol.* 11:R14. 10.1186/gb-2010-11-2-r14 PMC2872874 20132535

[B76] Zeng H. Wang G. Hu X. Wang H. Du L. Zhu Y. (2013). Role of microRNAs in plant responses to nutrient stress. *Plant Soil* 374 1005–1021. 10.1007/s11104-013-1907-6

[B77] Zeng H. Q. Wang G. P. Zhang Y. Q. Hu X. Y. Pi E. X. Zhu Y. Y. (2016). Genome-wide identification of phosphate-deficiency-responsive genes in soybean roots by high-throughput sequencing. *Plant Soil* 398 207–227. 10.1007/s11104-015-2657-4

[B78] Zhang B. (2015). MicroRNA: a new target for improving plant tolerance to abiotic stress. *J. Exper. Bot.* 66 1749–1761. 10.1093/jxb/erv013 25697792 PMC4669559

[B79] Zhang G. Yin S. Mao J. Liang F. Zhao C. Li P. (2016). Integrated analysis of mRNA-seq and miRNA-seq in the liver of *Pelteobagrus vachelli* in response to hypoxia. *Sci. Rep.* 6:22907. 10.1038/srep22907 PMC4785494 26961594

[B80] Zhang J. H. Huang M. H. Liang J. S. Pan Y. Cheng L. J. Wu J. (2016). Genome-wide mining for microRNAs and their targets in *Betula luminifera* using high-throughput sequencing and degradome analyses. *Tree Genet. Genom.* 12:99.

[B81] Zhang J. H. Wu T. Li L. Han S. Y. Li X. M. Zhang S. G. (2013). Dynamic expression of small RNA populations in larch (*Larix leptolepis*). *Planta* 237 89–101. 10.1007/s00425-012-1753-4 22983700

[B82] Zhang S. Sun L. Kragler F. (2009). The phloem-delivered RNA pool contains small noncoding RNAs and interferes with translation. *Plant Physiol.* 150 378–387. 10.1104/pp.108.134767 19261735 PMC2675743

[B83] Zhao M. Ding H. Zhu J. K. Zhang F. Li W. X. (2011). Involvement of miR169 in the nitrogen-starvation responses in *Arabidopsis*. *New Phytol.* 190 906–915. 10.1111/j.1469-8137.2011.03647.x 21348874 PMC3586203

