# Supplementary figures and images for: Profiling of MicroRNAs and Their Targets in Roots and Shoots Reveals a Potential MiRNA-Mediated Interaction Network in Response to Phosphate Deficiency in the Forestry Tree Betula luminifera
Source: Front Genet. 2021 Jan 28;12:552454. doi: 10.3389/fgene.2021.552454 (PMC7876418; doi:10.3389/fgene.2021.552454)

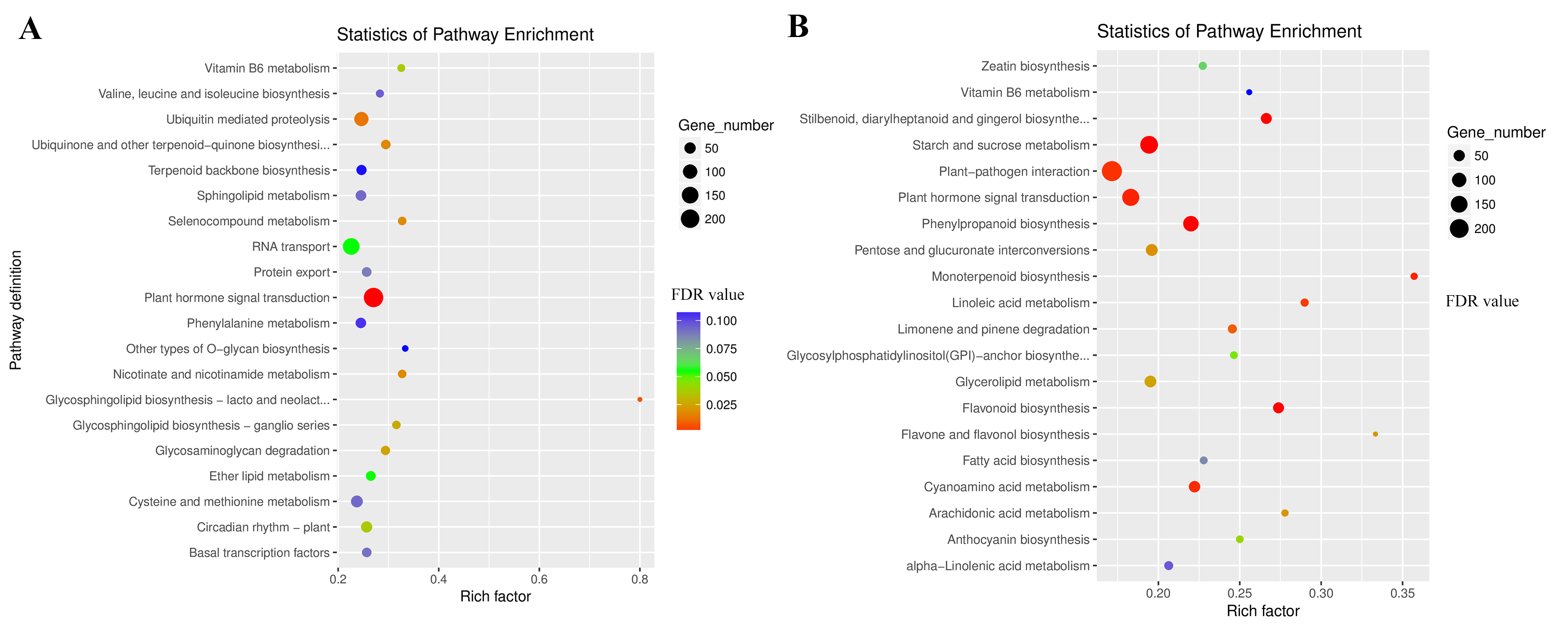

Supplement: Supplementary Figure 1 — KEGG pathway enrichment analysis of DE mRNAs under −Pi stress. The 20 most-enriched KEGG pathways among the DE mRNAs identified in (A) roots and (B) shoots under −Pi stress using KOBAS (2.0) with FDR ≤ 0.05. [file Image_1.TIF]

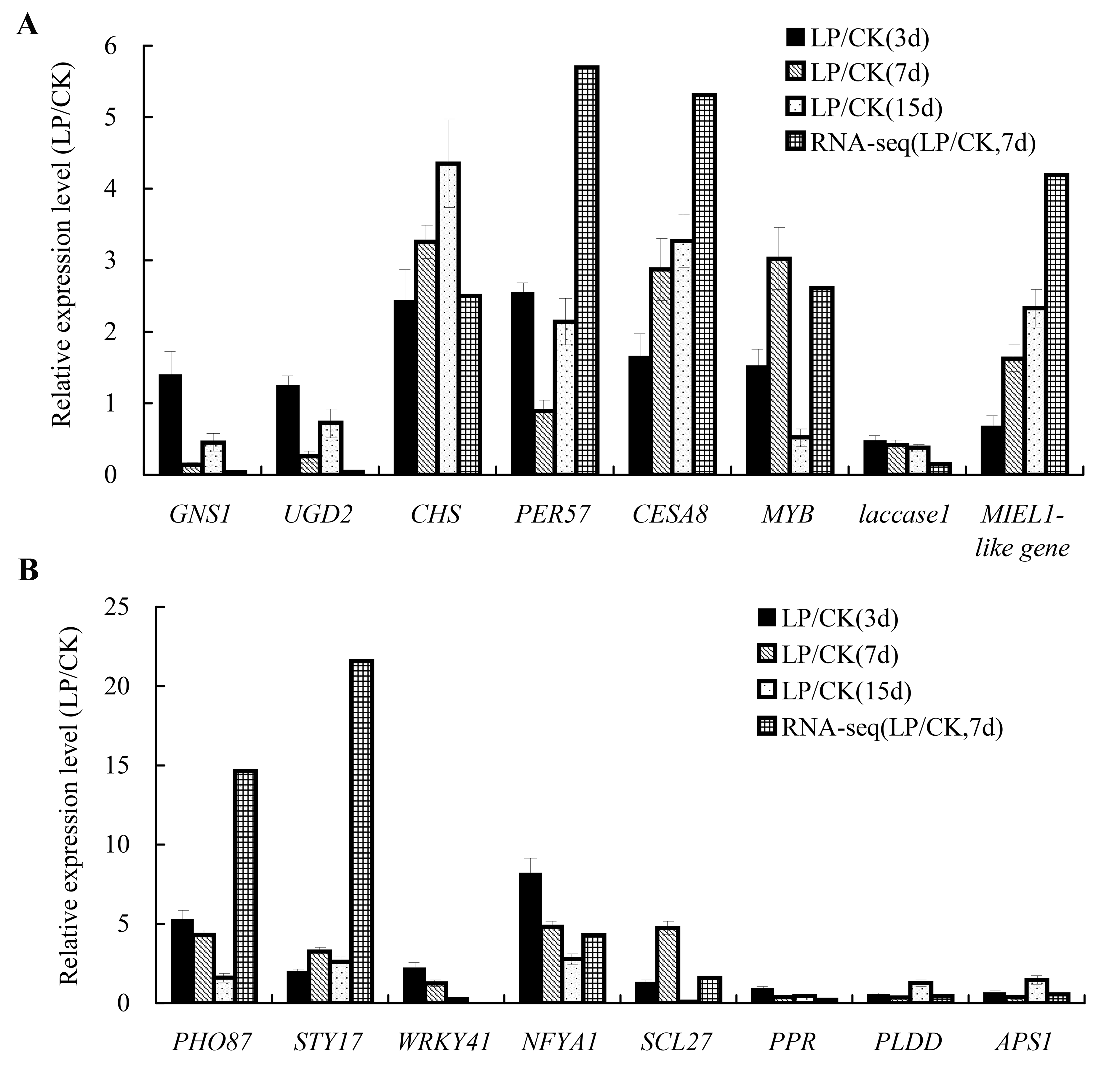

Supplement: Supplementary Figure 2 — Validation of the expression profiles of genes in B. luminifera shoots and roots identified by RNA-seq using qRT-PCR. (A) Relative expression of eight LP-responsive genes in shoots after 3, 7, and 15 days of LP treatment; (B) Relative expression of eight LP-responsive genes in roots after 3, 7, and 15 days of LP treatment. Expression level is represented by the ratio of LP treatment to CK. The value of relative expression level above one indicates that genes are upregulated by Pi starvation, and the value below one shows that genes are downregulated by Pi starvation. All expression levels were normalized to TUA and MDH in shoots and roots, respectively. The experiments were repeated three times. Error bars indicate standard deviation. [file Image_2.TIF]

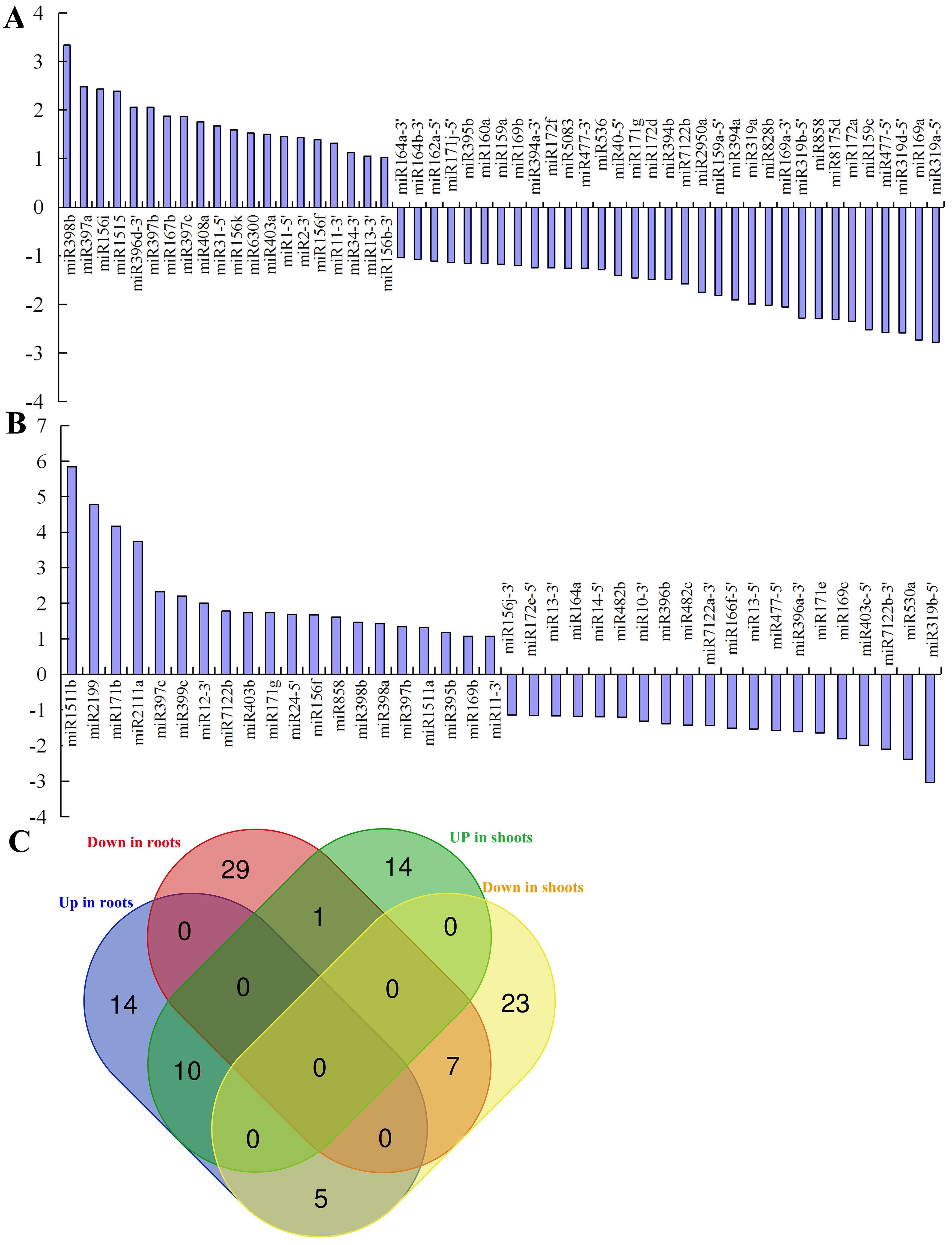

Supplement: Supplementary Figure 3 — Identification of DE miRNAs in shoots and roots after 7 days of Pi starvation. DE miRNAs in response to 7 days of Pi starvation in (A) shoots and (B) roots were identified using Fisher’s exact test, with the significance threshold set to 0.05. Only significantly differentially expressed miRNAs with a fold change greater than 2 are shown. (C) Venn diagrams showing the numbers of common DE miRNAs and the overlapping sets obtained across four comparisons. [file Image_3.TIF]

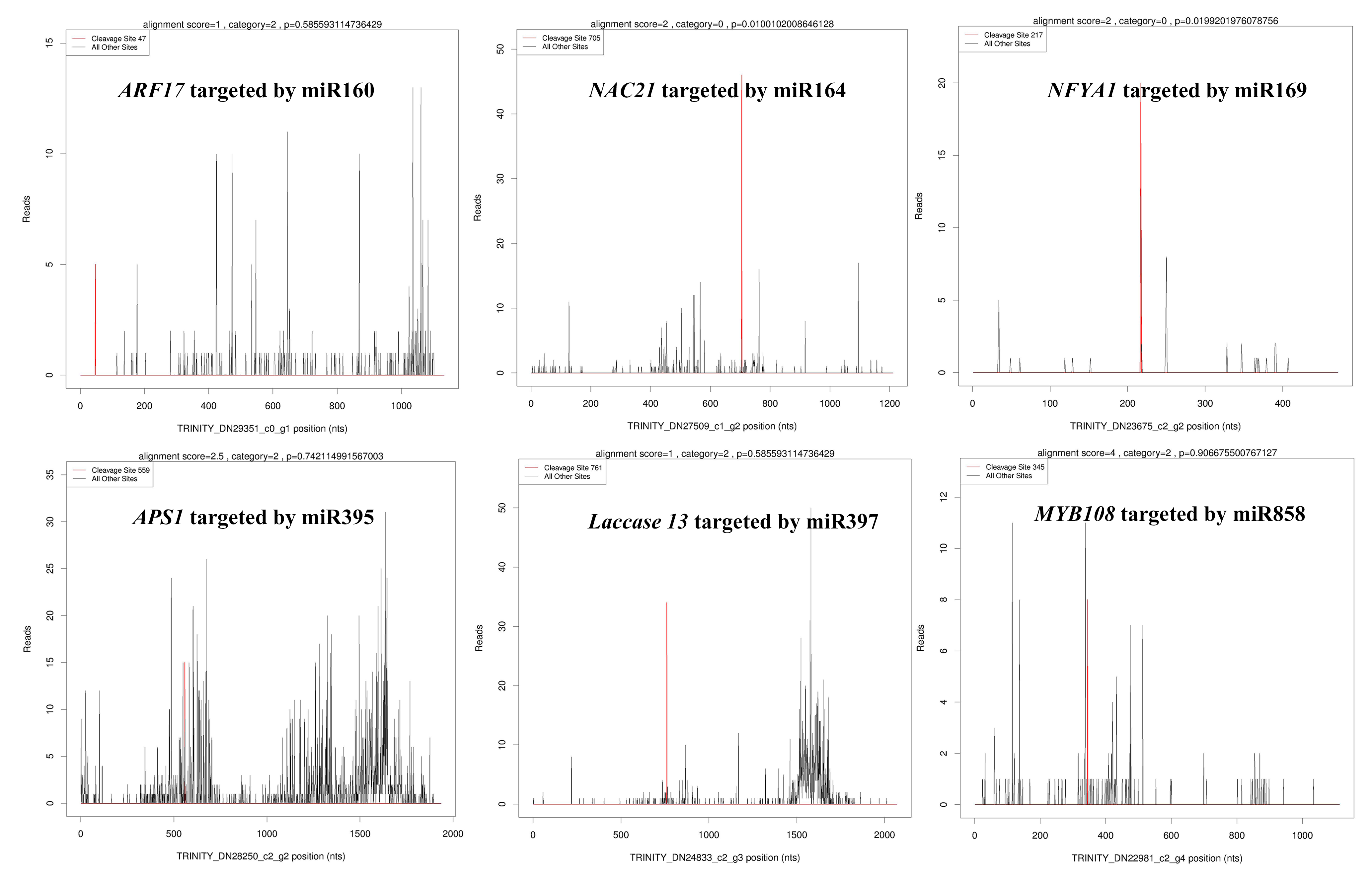

Supplement: Supplementary Figure 4 — Validation of miRNA target genes. Target plots (t-plots) of representative miRNA targets confirmed by degradome-Seq. The abundance of each signature is plotted as a function of its position in the transcript. The representative miRNAs and their corresponding targets are shown; the red line indicates the cleavage site of each transcript. ARF17, auxin response factor 17; NAC21, (NAM, ATAF, CUC 21); NFYA1, nuclear transcription factor Y subunit A1; APS1, ATP sulfurylase 1; MYB108, Myb domain protein 108. [file Image_4.TIF]

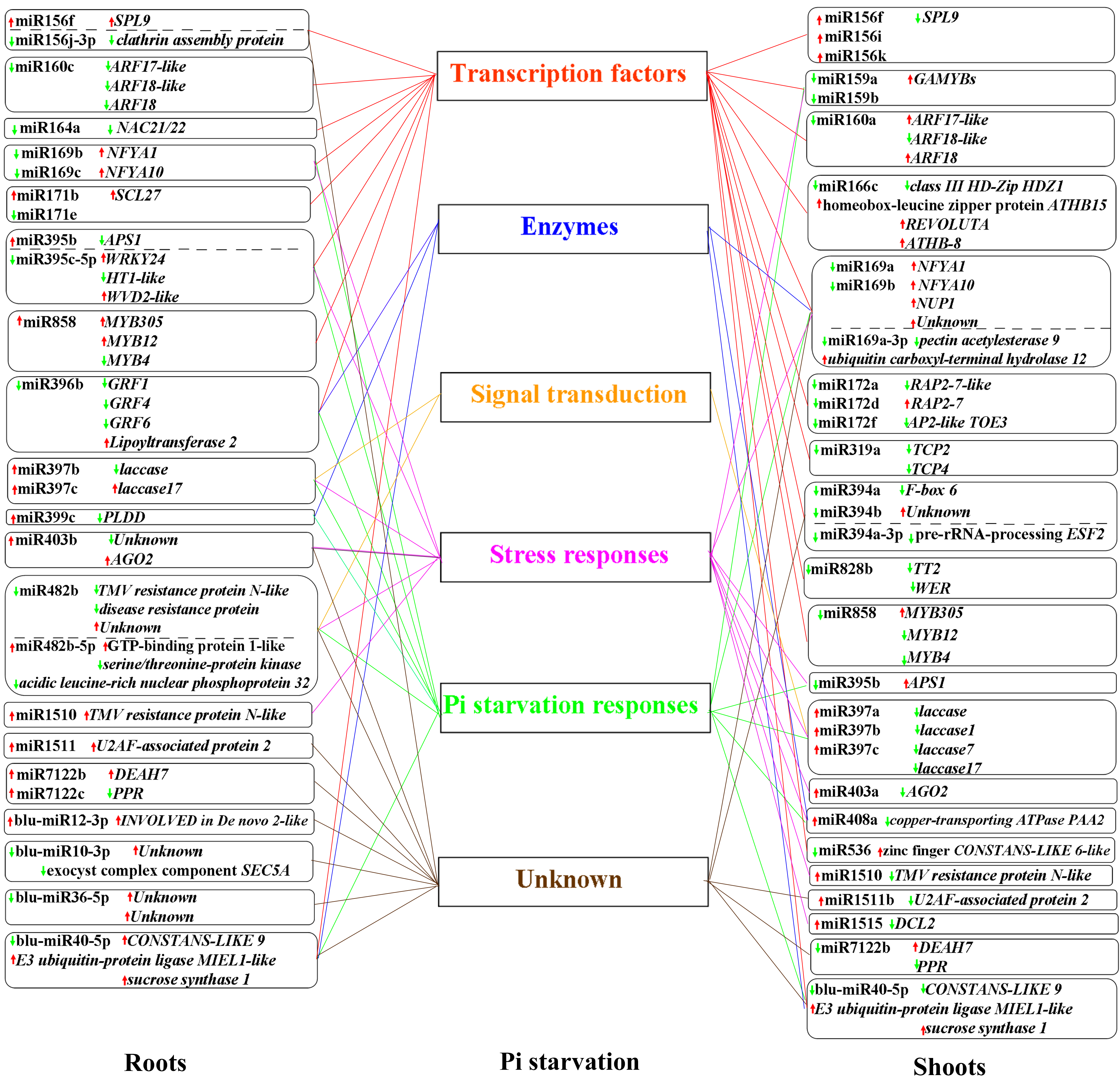

Supplement: Supplementary Figure 5 — Possible functional networks for −Pi-responsive miRNAs and their corresponding target genes in B. luminifera. Relationships between −Pi-responsive miRNAs and their target genes shown based upon putative physiological functions. Red arrows represent upregulation, whereas green arrows represent downregulation. [file Image_5.TIF]
