# Supplementary material for: Profiling of MicroRNAs and Their Targets in Roots and Shoots Reveals a Potential MiRNA-Mediated Interaction Network in Response to Phosphate Deficiency in the Forestry Tree Betula luminifera
Source: Front Genet. 2021 Jan 28;12:552454. doi: 10.3389/fgene.2021.552454 (PMC7876418; doi:10.3389/fgene.2021.552454)
Supplement: Supplementary Table 1 — 5′RLM-RACE primers for validation of miRNA targets. [file Table_1.DOC]

Table S1 5'RLM-RACE primers for validation of miRNA targets.

| Primer Name | Sequence (5’ to 3’) | Target information | Tm ℃ |
| --- | --- | --- | --- |
| T164-1 | 5′-ACTAATGATTTTCGTAATTGGCTTGGCG-3′ | miR164 target: *NAC21* | 69.3 |
| T169-1 | 5′-GGGCTGAACGGAAGAGAAATGTATGAAC-3′ | miR169 target: *NFYA1* | 68.7 |
| T169-2 | 5′-GAAATAGGCTTGTTCCTCGGCTACTCCA-3′ | miR169 target:  *NFYA10* | 70.2 |
| T397-1 | 5′-GCAGGGAGCAGATTTGTATTCAAGGATT-3′ | miR397 target:  *Laccase13* | 68.4 |
| T397-2 | 5′-TTAGAAAAACATTTGTGGTCTGTCCTGGG-3′ | miR397 target:  *Laccase17* | 68.9 |
