# Supplementary material for: Profiling of MicroRNAs and Their Targets in Roots and Shoots Reveals a Potential MiRNA-Mediated Interaction Network in Response to Phosphate Deficiency in the Forestry Tree Betula luminifera
Source: Front Genet. 2021 Jan 28;12:552454. doi: 10.3389/fgene.2021.552454 (PMC7876418; doi:10.3389/fgene.2021.552454)
Supplement: Supplementary Table 2 — Gene primers used in qRT-PCR. [file Table_2.DOC]

Table S2. Gene primers used in qRT-PCR.

| **Target genes** | **Gene specific forward primer**  **5’ to 3’** | **Gene specific reverse primer**  **5’ to 3’** |
| --- | --- | --- |
| *GNS1* | q*GNS1*-F: GTTTATTTGGAAGAAAGAGCCC | q*GNS1*-R: ATTGTTGTATCTGAGACTGGTTG |
| *UGD2* | q*UGD2*-F: CTCTATGTCGTCTCCACCACC | q*UGD2*-R: TTCTTTCTCCTGTTATCCCGC |
| *CHS* | q*CHS*-F: ACCCAAAGAGAACACCCCAGT | q*CHS*-R: ATGAATCTTTCTCGCCAATCG |
| *PER57* | q*PER57*-F: TCTGCTTGAGGACAGGTGGAT | q*PER57*-R: AGTTGATACTCTGGATGGGGG |
| *CESA8* | q*CESA8*-F: ATGGCTCCCACATAAATAACT | q*CESA8*-R: GCCTCTGTCTTCTCTCTTCTT |
| *GAMYB* | q*GAMYB*-F:AGTGCTAATGTTGGAAACGGAT | q*GAMYB*-R: ACTTCAAAGCCCCAGATGTAGG |
| *laccase 1* | q*laccase*-F: CTCCACCGTCACCACTCGC | q*laccase*-R: TCAATGGGACTTTGGCAGG |
| *MIEL1-like* | q*MIEL1*-F: CCTCCCTCTCCTTTTTTTCGTT | q*MIEL1*-R: ACAGCCAGCACTCACTGAAGAT |
| *PHO87* | q*PHO87*-F: ACCGATGAGTGATGCTGGAAT | q*PHO87*-R: GCCAACCCCACATCCATCT |
| *STY17* | q*STY17*-F: AGCATCTCCTTATTGACACGC | q*STY17*-R: AATGCCATAAAAATACCGACTG |
| *WRKY41* | q*WRKY41*-F: ACCCTTATTCCCTTTGATGACA | q*WRKY41*-R: GCCACCAGTTCTCCTATGCTA |
| *NFYA1* | q*NFYA1*-F: AAATGAAGTTGCTGAAGGTGAA | q*NFYA1*-R: GGCTGAACGGAAGAGAAATGTA |
| *SCL27* | q*SCL27*-F: GGAGGGCGTGTTGTCTGAGT | q*SCL27*-R: AAATCGGGAGAGGGTAGGGG |
| *PPR* | q*PPR*-F: AGGTGGATTTCGGCTTCTCTAT | q*PPR*-R: CAATTTTACCCTCAAGACAGAGC |
| *PLDD* | q*PLDD*-F: GCCAGCACACAAGTGACAGATG | q*PLDD*-R: CGATAAGACCTCCCACGACAA |
| *APS1* | q*APS1*-F: TCCCAATCAGAAATCCATACAT | q*APS1*-R: CTATGACAGTTTGGTTCCCGC |
| *TUA* | q*TUA*-F: ACTAACCTTGTACCATATCCTCGT | q*TUA*-R: GGCTCAAATACTGCACTTGTGA |
| *MDH* | q*MDH*-F: CTCATGCCTCACTTGAATGCTCGT | q*MDH*-R: AGCTCCTCCATCATTAACAGTTCC |
