# Supplementary material for: Profiling of MicroRNAs and Their Targets in Roots and Shoots Reveals a Potential MiRNA-Mediated Interaction Network in Response to Phosphate Deficiency in the Forestry Tree Betula luminifera
Source: Front Genet. 2021 Jan 28;12:552454. doi: 10.3389/fgene.2021.552454 (PMC7876418; doi:10.3389/fgene.2021.552454)
Supplement: Supplementary Table 3 — miRNA primers used in qRT-PCR. [file Table_3.DOC]

Table S3. miRNA primers used in qRT-PCR.

MRQ 3′ primerNCode™ from Mir-X^TM^ miRNA First-Strand Synthesis Kit (TaKaRa) was used as reverse primer.

| miRNA | Forward primer (5′→ 3′) | Length |
| --- | --- | --- |
| blu-miR159a | TTTGGATTGAAGGGAGCTCTA | 21 |
| blu-miR164a | TGGAGAAGCAGGGCACGTGCA | 21 |
| blu-miR169b | TGAGCCAAGAATGACTTGCC | 20 |
| blu-miR169c | TGAGCCAAGAATGACTTGCCGAC | 23 |
| blu-miR171e | TTGAGCCGTGCCAATATCACA | 21 |
| blu-miR395b | CTGAAGTGTTTGGGGGAACTC | 21 |
| blu-miR397a | TCATTGAGTGCAGCGTTGATG | 21 |
| blu-miR398b | TTGTGTTCTCAGGTCACCCCT | 21 |
| blu-miR399c | TGCCAAAGGAGAATTGCCC | 19 |
| blu-miR482b | TCTTCCCTACTCCGCCCATGCC | 22 |
| blu-miR530a | TCTGCATTTGCACCTGCACCT | 21 |
| blu-miR828b | TCTTGCTCAAATGAGTATTCCG | 22 |
| blu-miR858 | TTCGTTGTCTGTTCGACCTTG | 21 |
| blu-miR7122b | TTATTCAGAGAAATCACGGT | 20 |
| blu-miR11-5' | AAATTCATCCATCTTTGTTCATTT | 24 |
| blu-miR13-3' | GCCATTTCAATGTGAAAAAGGATT | 24 |
| blu-miR40-5' | AGAAAAGAAAAGGGAAGGATT | 21 |
| qU6-F | TCGGGGACATCCGATAAAATTGGAA | 25 |
| qU6-R | GGACCATTTCTCGATTTATGCGTGTCA | 25 |
