# Supplementary material for: Profiling of MicroRNAs and Their Targets in Roots and Shoots Reveals a Potential MiRNA-Mediated Interaction Network in Response to Phosphate Deficiency in the Forestry Tree Betula luminifera
Source: Front Genet. 2021 Jan 28;12:552454. doi: 10.3389/fgene.2021.552454 (PMC7876418; doi:10.3389/fgene.2021.552454)
Supplement: Supplementary Table 4 — tRNA identification in sRNA libraries. [file Table_4.DOC]

Table S4. tRNA identification in sRNA libraries.

| length | CK_R | ratio (%) | CK_SL | ratio (%) | LP_R | ratio (%) | LP_SL | ratio (%) |
| --- | --- | --- | --- | --- | --- | --- | --- | --- |
| 18 | 27726 | 8.42 | 30668 | 18.28 | 41808 | 27.72 | 2734 | 2.50 |
| 19 | 46981 | 14.27 | 72243 | 43.07 | 43119 | 28.59 | 7128 | 6.51 |
| 20 | 31013 | 9.42 | 12817 | 7.64 | 19628 | 13.01 | 5172 | 4.72 |
| 21 | 30620 | 9.30 | 13684 | 8.16 | 13698 | 9.08 | 9601 | 8.77 |
| 22 | 28616 | 8.69 | 8639 | 5.15 | 11211 | 7.43 | 7052 | 6.44 |
| 23 | 39427 | 11.98 | 10892 | 6.49 | 8970 | 5.95 | 11887 | 10.85 |
| 24 | 37924 | 11.52 | 8076 | 4.81 | 5811 | 3.85 | 15390 | 14.05 |
| 25 | 49947 | 15.17 | 6266 | 3.74 | 5160 | 3.42 | 20803 | 18.99 |
| 26 | 18922 | 5.75 | 2580 | 1.54 | 953 | 0.63 | 13288 | 12.13 |
| 27 | 11312 | 3.44 | 1175 | 0.70 | 289 | 0.19 | 8142 | 7.43 |
| 28 | 6697 | 2.03 | 706 | 0.42 | 179 | 0.12 | 8325 | 7.60 |
| total | 329185 |  | 167746 |  | 150826 |  | 109522 |  |
